# Comparative Analysis of Lifecycle-Dependent A to I RNA Editing in Three Members of the *Microbotryum violaceum* Fungal Complex

**DOI:** 10.3390/jof12090641

**Published:** 2026-08-28

**Authors:** Shikhi Baruri, Nasara Shamsudeen, Alycia C. R. Lackey, Joseph P. Ham, Michael H. Perlin

**Affiliations:** Department of Biology and Program on Disease Evolution, University of Louisville, Louisville, KY 40292, USA; shikhi.baruri@louisville.edu (S.B.); saeedatunasara.shamsudeen@louisville.edu (N.S.); alycia.lackey@louisville.edu (A.C.R.L.);

**Keywords:** A-to-I RNA editing, basidiomycetes, *Microbotryum*, transcriptomics, lifecycle regulation, phylogenetic comparison

## Abstract

A-to-I RNA editing is increasingly recognized as a regulator of fungal development and pathogenesis, yet its extent and lifecycle dependence remain poorly characterized in basidiomycetes. *Microbotryum superbum* (MvSup), *M. intermedium* (MI), and *M. lychnidis-dioicae* (MVLG) are members of the *M. violaceum* fungal complex. Each species infects specific host plant species, resulting in commonly anther-smut disease. The lifecycle of these basidiomycete fungi includes the haploid, mating, and infection stages. RNA editing is a post-transcriptional process where adenosine (A) is converted to inosine (I) by adenosine deaminase enzymes, and such modifications to RNAs may lead to synonymous and nonsynonymous codon changes, thereby altering protein function. Here, we compared A-to-I editing across the haploid, mating, and infection stages of these three related species to determine how editing patterns vary with lifecycle stage and among closely related fungal pathogens. The a2 haploid strain of MI had fewer editing sites compared to other haploid strains. The predicted codon/amino acid changes in each haploid strain across the three species indicated three primary types of resulting amino acid substitutions that were common to both of the mating-type strains across the three species: threonine to alanine, lysine to glutamic acid, and valine to alanine. During the mating stage of MvSup, a synonymous codon change was found in a mitogen-activated protein kinase domain-containing protein within the protein’s conserved region. Gene expression analysis revealed that certain genes, uniquely edited during the mating stage of MvSup, tend to be upregulated in the haploid stage but downregulated during mating, and vice versa. Research on RNA editing in basidiomycetes is relatively new. RNA editing mechanisms in fungi have been implicated in fungal pathogenesis, although the exact roles and implications remain unclear. Additional research will help us understand the functional significance of this apparently ubiquitous process in several members of the *Microbotryum* fungal complex, with possible ramifications more generally in fungi.

## 1. Introduction

The control of gene expression and the functional diversity of RNA molecules are significantly influenced by the post-transcriptional process known as RNA editing. One well-known type of RNA editing is mediated by adenosine deaminases acting on RNA, which facilitate the conversion of adenosine (A) to inosine (I) within RNA molecules. Regulation of gene expression has been demonstrated to be functionally affected by this mechanism [1,2]. This kind of RNA alteration can cause nonsynonymous codon changes that may have an impact on the function of the encoded protein. A-to-I RNA editing of mRNAs has been investigated in mammals for more than 30 years, but it was just recently found in fungi [3]. So far, studies on RNA editing in fungi have demonstrated that A-to-I RNA editing occurs widely in ascomycete fungi and is not dependent on adenosine deaminases acting on RNA (ADAR) enzymes [4]. Rather, the reports so far implicate adenosine deaminases acting on tRNA (ADAT) enzymes [4,5,6]. Interestingly, enzymes referred to as ADATs in ascomycetes have been shown to act also on mRNAs [4,7]. Although there have been some reports of RNA editing in Basidiomycetes, the confidence that these observations rigorously demonstrate such RNA modifications in basidiomycete species has been limited [8]. In ascomycete fungi like *Neurospora crassa*, A-to-I RNA editing has been shown to be developmentally controlled and adapted for sexual reproduction [5]. Furthermore, it has been suggested in some studies that A-to-I RNA editing of certain genes may be crucial for the autophagy function during sexual reproduction [9]. A-to-I editing in fungi, such as *Fusarium graminearum*, as mentioned above, has been demonstrated to be independent of ADAR enzymes that are typically present in mammals [4]. A significant proportion of pseudogenes are edited to eliminate stop codons, with the majority of the editing process affecting coding areas, which result in amino acid alterations in proteins [4], a common phenomenon in fungi [10]. A-to-I RNA editing has been shown to provide adaptive benefits in fungi, especially in *Fusarium* species, for resolving survival–reproduction trade-offs. The significance of this method in fungal biology is shown by the abundant A-to-I editing sites found in the perithecia of *F. graminearum* [11]. Additionally, experimental validation of the adaptive benefit and functional importance of A-to-I RNA editing in fungi highlights the role this mechanism plays in fungal gene regulation and evolution.

A group of over eighty-nine basidiomycete fungal species known as the *Microbotryum violaceum* complex infects a similarly large group of plant host species (https://www.inaturalist.org/taxa/319843-Microbotryum; accessed on 25 May 2025) [12] that reflect phylogenetic patterns of their caryophyllaceous hosts [12,13]. Each species in this complex shows significant post-zygotic isolation, which is evidence of great genetic differentiation and specialization [14,15]. The complex has been found to infect over 100 species in the Caryophyllaceae family, and based on patterns of host specificity and morphological variation, it is considered to be a species complex [14,16].

The research on A-to-I RNA editing in ascomycete fungi has revealed its widespread occurrence and developmental regulation, particularly in the context of sexual reproduction. Here, we focus on three members of the *Microbotryum violaceum* complex: *Microbotryum superbum* (MvSup), *M. intermedium* (MI), and *M. lychnidis-dioicae* (MVLG). These three species provide an excellent opportunity to understand the molecular processes regarding sexual reproduction, host preference, and adaptability. Understanding how A-to-I RNA editing happens in these fungi could potentially elucidate the genetic and molecular basis of reproductive isolation and speciation in this complex [17,18].

The members of the *Microbotryum violaceum* complex have different stages of their life cycle, including the haploid, mating, and infection stages. The infection process starts when diploid teliospores germinate on an appropriate plant surface. Following meiosis, these spores produce linear tetrads of haploid basidiospores that reproduce via budding to yield yeast-like sporidial cells. Such cells can mate with partners of opposite mating type. After developing conjugation bridges, mated cells differentiate into dikaryotic hyphae that can penetrate the host tissue after receiving appropriate cues from the host plant. The infection becomes systemic, and the hyphae proceed toward the floral primordia. After this stage, nuclear fusion takes place, and the hyphae develop into diploid teliospores. Finally, these teliospores replace the pollen in the anthers of the flowers. The cycle is repeated through transmission by pollinators who spread the spores to uninfected flowers [19].

In this paper, we examined A-to-I RNA editing for MvSup, MI, and MVLG in three different stages of their lifecycles to understand how A-to-I RNA editing varies among these members of the fungal species complex. Our research aims to identify the commonalities as well as distinctive variations in RNA editing within this particular fungal group. Additionally, in Basidiomycota, RNA editing may be required to grow in a different environment or substrate, a feature possibly associated with fungal pathogenesis, although the effect on protein functions after editing is still unclear [20]. Thus, our research should lead to a better understanding of how the editing process influences the expression of pathogenicity-related genes in these fungi. With this information, strategies could be developed to prevent or lessen the effects of fungal diseases on hosts, which is crucial for managing ecosystems and agriculture. Furthermore, we want to explore both the differences and commonalities in RNA editing between ascomycetes and basidiomycetes, such as *Microbotryum*, during sexual developmental stages of their life cycles.

## 2. Materials and Methods

**Figure 1:**
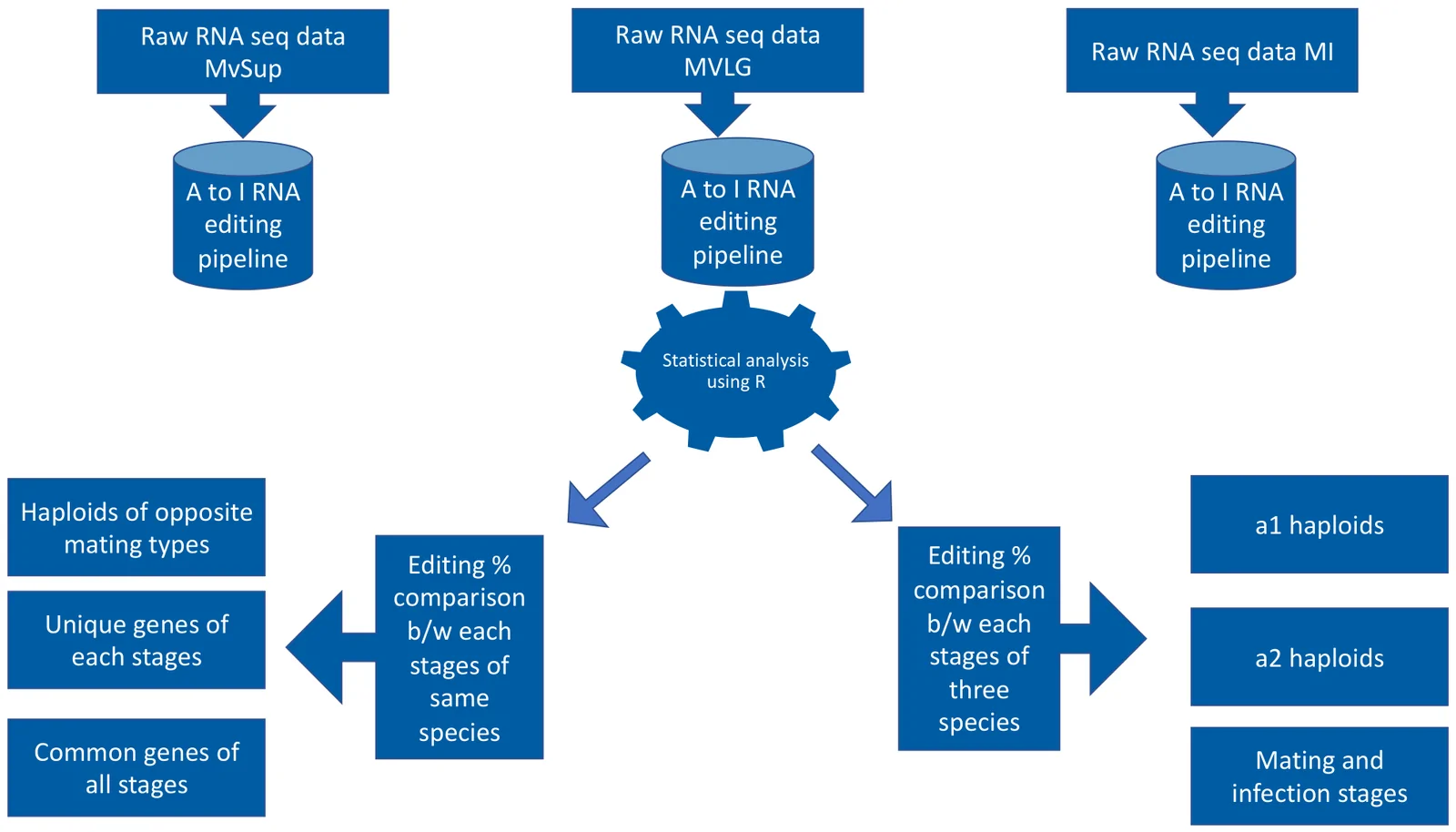
The overall approach to compare RNA editing sites across the species and stages of the *Microbotryum* life cycle. We illustrate the data used from three species and the two types of comparisons made within and between species. R software, version 2022.12.0+353, was used in the overall analysis.

Inspection of the respective genomes of *Microbotryum* species examined here (MvSup, MI, and MVLG) revealed that each contains orthologues of both ADAR and ADAT family proteins. MvSup contains two putative ADAR-like, MvSupADA2 and MvSupADA3, and one predicted ADAT-like, MvSupADA1. Similarly, MVLG contains orthologues of these putative ADARs, MVLG_03062 and MVLG_01566, and a putative ADAT, MVLG_05607. Finally, MI is predicted to have two ADAR orthologues, BQ2448_2751-1 and BQ2448_904-1, as well as a predicted ADAT, BQ2448_2453-1. Protein modeling using NCBI CDS Domain Identifier (https://www.ncbi.nlm.nih.gov/Structure/cdd/wrpsb.cgi; accessed 23 May 2025) supported the characterization of the proteins MvSupADA2 and MvSupADA3 of MvSup, and their orthologues in MVLG, i.e., MVLG_03062 and MVLG_01566, respectively, as putative members of the ADAR family (Baruri et al., in preparation). Phylogenetic analysis further showed that the *Microbotryum* candidate deaminases are closely related within the genus but did not confidently resolve them as orthologues of either canonical ADAR or ADAT proteins. Therefore, the terms “ADAR-like” and “ADAT-like” are used here as provisional descriptions based on sequence and domain similarity. Moreover, both MvSupADA2 and MVLG_01566 are upregulated in the late stage of infection in male plants (Supplementary Table S1). As these proteins are candidate adenosine deaminases potentially associated with A-to-I changes, we focused here on such RNA editing, irrespective of the specific adenosine deaminase(s) responsible in these species. In this paper, A-to-I editing sites of RNA were identified and compared across different species. In the three species, their comparison was performed in two stages—two haploid stages with different mating types and a mating stage. In two species, the infection stages were also compared. Within species, all stages were compared to identify commonly edited genes. The RNA-edited genes in just one of its stages but not in the other stages were considered stage-specific or uniquely edited genes (Figure 1).

**Figure 2:**
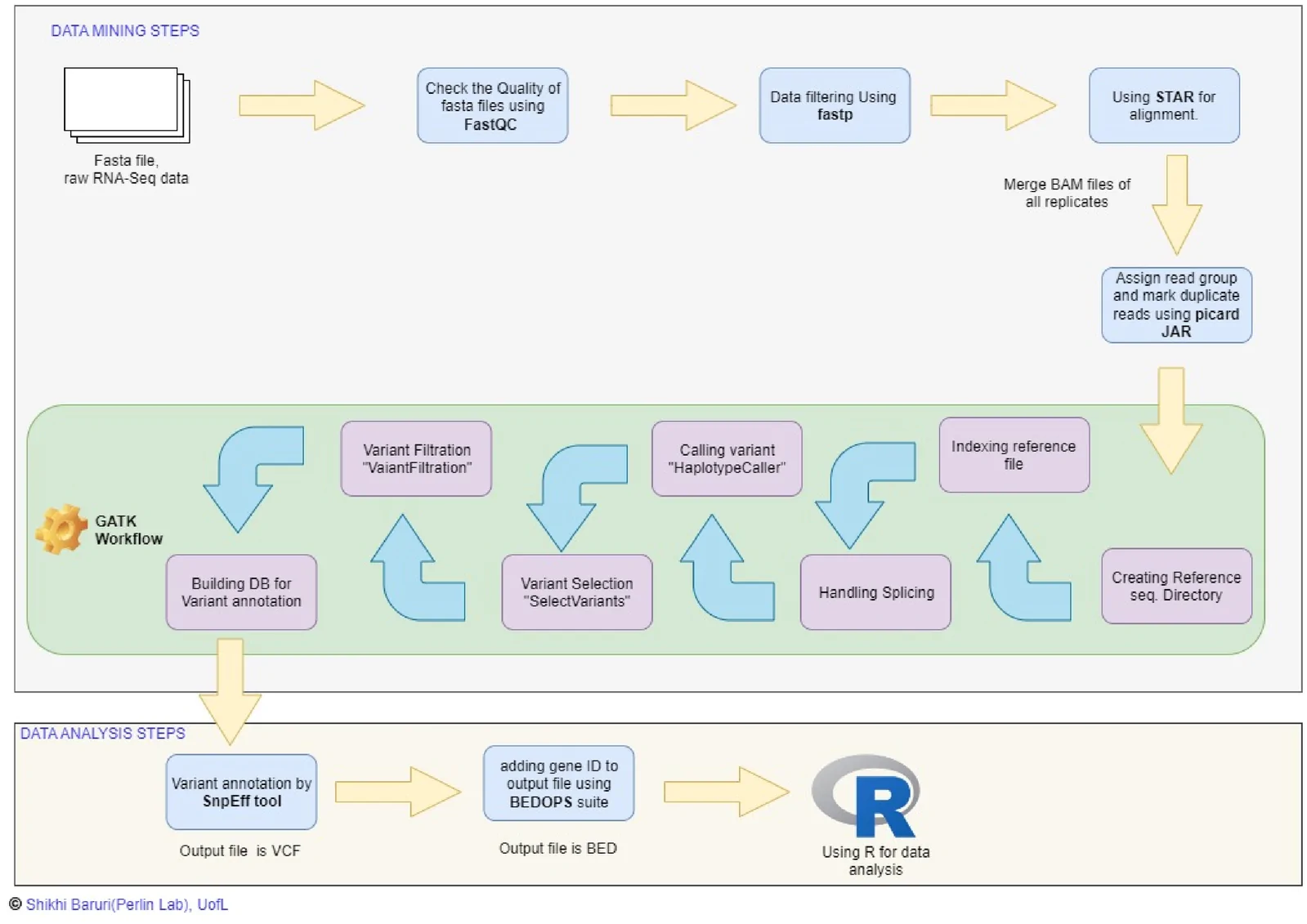
Pipeline to identify RNA editing sites.

The process of identifying RNA editing sites is multi-step and primarily focuses on RNA sequencing data processing. This includes two basic steps: data mining and data analysis. The detailed workflow is shown in Figure 2. For the data mining step, the GATK Best Practices workflows were used, which are highly regarded in the field and a gold standard [21]. Relevant customized codes are available at the following link: https://github.com/shikhi18/RNAEDITING (accessed 23 April 2025).

### 2.1. RNASeq

In this study, RNA was extracted from several stages of fungal development and the fungal lifecycle: the haploid, yeast-like stage; during mating; and during active infection of the host plant at several stages. For MVLG p1A1 and p1A2, RNA growth conditions, RNA isolation, and RNASeq were as described previously [22]. RNASeq was completed at the Broad Institute (Cambridge, Massachusetts), and we purified polyA RNA and constructed a strand-specific library for each sample. Samples (3 biological replicates each) were sequenced with Illumina technology, generating 76-base paired reads. For the rich and nutrient-limited samples, 90% or 89% of reads aligned, respectively, to the reference genome for MVLG p1A1; for the infected male buds, only 23% of reads aligned. This was expected, as these samples also contain the host *Silene* RNAs. For MI and MvSup, strains were similarly grown on potato dextrose agar (PDA), and for haploid samples or compatible mating-type strains for each species, they were mixed and applied to water agar plates. Buds from infected *D. pavonius* were used for RNASeq of MvSup. Three biological replicates were used for each sample for RNA extraction. Total RNA was isolated using the Qiagen RNeasy Plant Mini Kit (Qiagen, Valencia, CA, USA). After total RNA isolation, several quality control measures were taken. Concentration and purity were assessed using a NanoDrop 2000 spectrophotometer (Thermo Scientific, Waltham, MA, USA); 260/280 and 260/230 ratios > 1.8 were considered satisfactory for RNAseq application. Additionally, cDNA was prepared and used as a template for intron-spanning primers in PCR reactions to verify the lack of genomic DNA contamination. Bioanalyzer analysis was completed to detect intact 18S and 23S RNA as a measure of overall RNA quality. After passing all quality control measurements, RNA samples from MI and MvSup were sent for sequencing to CD Genomics (Shirley, NY, USA). Paired-end RNAseq of three biological replicates per condition (mated, haploid a_1_ and haploid a_2_, and in planta) was performed. While the library for MI was strand-specific, that for MvSup was not. Each RNASeq sample for these was sequenced to provide a depth of 20 million reads/sample. A table (Supplementary Table S2; [22,23,24]) with accession numbers and descriptions of strains/growth conditions for all RNA-seq datasets generated or used in this study is provided.

### 2.2. Quality Control of Raw Data

After obtaining the raw data (fastq files) from CD Genomics (Shirley, NY, USA) or investigators, the quality of the original reads, including sequencing error rate distribution and GC content distribution, was evaluated using FastQC, v 0.12.0. The original sequencing data contained low-quality reads and adapter sequences. To ensure the quality of data analysis, raw reads were filtered to get trimmed reads, and the subsequent analysis was done based on such reads. Raw sequencing reads were quality-filtered and trimmed using fastp (v0.23.4) [25] with the following parameters: low-quality bases were trimmed from both ends using a sliding window of 4 bases with a minimum average quality score of 20. Reads shorter than 30 bp after trimming were discarded. Adapter sequences were detected and removed automatically by fastp. For RNAseq, we have used three biological replicates for each haploid strain of the three species.

### 2.3. Alignment to Reference Genome

In this step, the genome annotation files for MI and MVLG were downloaded from Ensemble Fungi (https://fungi.ensembl.org/index.html; accessed 23 May 2025). For MvSup, the genome annotation file was provided by R. Rodriguez de la Vega and T. Giraud [24]. The gff file was converted into a gtf file using the cufflinks package, and then the STAR genome index was created. After the 2-pass mapping procedure, the STAR tool was used with default parameters for the read mapping on the reference genome [26].

### 2.4. Post-Alignment Processing Using GATK (GATK Best Practices)

After post-alignment, sorting was done using Samtools, version 1.24 [27]. Picard (http://broadinstitute.github.io/picard/; accessed 23 May 2025) was used to assign the read group (RG) tag and mark duplicates. The reference sequence dictionary was created, and the reference file was indexed. The SplitNCigarReads step was used to rewrite reads into a format more amenable to variant calling. This format splits reads into exon segments (getting rid of Ns) and hard-clips any sequences overhanging into the intronic regions. In the variant calling step, GATK’s HaplotypeCaller function was used for calling variants in GVCF mode to call variants from RNA-Seq data [28]. This step is vital for identifying potential RNA editing sites. After that, the “Select Variants” and “Variant Filtration” functions were used for variant selection. To eliminate low-confidence variants, raw SNPs were processed using the GATK (v4.3.0.0) VariantFiltration tool. The applied filtering criteria included: quality by depth (QD) less than 2.0, variant quality (QUAL) below 30.0, strand odds ratio (SOR) exceeding 3.0, Fisher strand bias (FS) greater than 60.0, mapping quality (MQ) under 40.0, mapping quality rank sum (MQRankSum) less than −12.5, and read position rank sum (ReadPosRankSum) below −8.0. Variants that did not meet any of these thresholds were marked in the output VCF file for further analysis.

In the Annotation step, we first built the database for variant annotation, and then the variants were annotated using the SnpEff tool version 5.4c, which provides information about each variant’s genomic context to understand the functional impact of RNA editing sites (https://gatk.broadinstitute.org/hc/en-us; accessed 23 May 2025). For increased stringency and more conservative estimates, the A-to-I editing sites were selected using a minimum threshold of 10% for the editing level. Genes identified as being edited frequently had editing levels as high as 100%. For example, the MVLG_05234 ABC transporter gene is edited at position 106520 to a level of 100% throughout each stage of the lifecycle examined. This indicates that all mRNA molecules for this gene experience A-to-I editing at position 106520 during the entire life cycle. Next, we converted the output vcf file into a bed file for statistical analysis. All statistical tests were performed using R software Version 2022.12.0+353 [29]. REDI tool version 1.2.1 was used to verify the comparison for all three species [30]. The nucleotide preferences surrounding the editing sites were visualized using WebLogo version 3 [31].

### 2.5. Growth Conditions and Genome Resequencing

Accurate identification of A-to-I RNA editing sites in bioinformatics requires using DNA re-sequencing data as a control in addition to RNA sequencing data. Previous studies highlighted that to acquire an accurate profile of RNA editing sites, it is necessary to combine a transcriptome with genomic DNA resequencing of a matched sample [32]. In this study, we used DNA sequencing data as a control to identify single-nucleotide polymorphism (SNP) sites in the genome of the strains used for RNASeq, compared with reference genomes. For these genome re-sequencing experiments, we used two replicates of each haploid mating-type strain of the three species for isolation of genomic DNA. The genomic DNA was extracted from both mating-type strains of the three species of *Microbotryum*, which were grown in YPD media overnight at 28 °C, followed by a PCI (phenol-chloroform-isoamyl alcohol) extraction protocol [19]. The extracted DNA was then washed with 70% ethanol. Finally, to remove any residual RNA contamination, we treated the genomic DNA with PureRec RNase A enzyme (Zymo Research, Irvine, CA, USA), and further purification was done with the genomic DNA clean and concentrator kit (Zymo Research) to ensure purity and quality. Then, two biological replicates of the genomic DNA of both mating-type strains, for each of the three species used for RNASeq, were re-sequenced using nanopore technology (Plasmidsaurus, Louisville, KY, USA). For the alignment of DNA seq data with the reference genome, the BWA-MEM2 tool was used [33,34,35]. Alignment with the respective reference genome allowed identification of the SNP sites in re-sequenced replicates; SNPs were also identified among replicates. The variation between re-sequenced replicates was the difference observed between re-sequenced replicates. Replicate SNPs were used to distinguish strain-specific variants from true sequencing/strain noise, and remaining SNPs between the two mating-type strains of each species were used to filter RNA editing candidates. The three re-sequenced genomes were submitted to NCBI and are available with the following Accession Numbers: *M. superbum* (WGS), PRJNA1201796; *M. lychnidis-dioicae* (WGS), PRJNA1202200; *M. intermedium* (WGS), PRJNA1202575. RNA was isolated from three species of *Microbotryum* cultivated under conditions outlined in our earlier studies [19,24]. In summary, haploid cells were grown on potato dextrose agar (PDA) at temperatures ranging from 22 °C to 28 °C. These cells were then collected for RNA extraction, utilizing either the Zymo RNA Extraction Kit (Zymo Rresearch. Irvine, CA, USA) or the Qiagen RNeasy Plant Mini Kit, based on the species and conditions. Prior to conducting mating experiments, the haploid cells were placed in distilled, sterilized water and adjusted to a density of 10^9^ cells/mL. These cells, representing the two distinct mating types (a1 and a2), were then mixed in equal amounts for the mating tests. This method ensured a consistent and high-density cell suspension to enhance mating efficiency during the 48 h incubation period on water agar Petri dishes at 13 °C. The success of the mating process was visually confirmed under a light microscope (Carl-Zeiss-Strasse 22, 73447 Oberkochen, Germany) by observing the formation of conjugation tubes between the cells [24].

After identification of SNPs between replicates of each genome, the sites from RNASeq data that matched SNPs found after DNA re-sequencing were eliminated from the inventory of RNA editing sites for each species/condition. In the case of MvSup, the RNA sequencing library was non-strand specific and constructed with the poly(A) method. Thus, it was not feasible to determine the strand where RNA editing took place. Therefore, for this species, we consider both the A-to-G and T-to-C base changes in positive- and negative-stranded genes, respectively [36]. To confirm this strand assignment, each candidate site’s substitution direction was cross-referenced against the strand of its overlapping gene in the genome annotation; only sites where the direction matched the gene’s strand (A-to-G for plus-strand genes, T-to-C for minus-strand genes) were retained as candidate editing sites.

### 2.6. Statistical Analysis

In each of the three species, the proportion of RNA editing sites was analyzed between haploid types. The estimated mean proportion of RNA editing sites between a1 and a2 mating types was calculated with 95% confidence intervals. Any interval not overlapping zero means the proportion of edits in a1 differs from a2 for all three species. Proportional differences within each species were determined based on z-score and *p*-value using the R package, version 2022.12.0+353 [29]. For each species, the proportion of editing events attributed to the a1 mating type was calculated by dividing the number of retained synonymous and nonsynonymous RNA editing events in the a1 mating type by the combined number of retained synonymous and nonsynonymous RNA editing events in both the a1 and a2 mating types. Only events remaining after quality filtering and genomic SNP filtering were included. Therefore, the denominator was the combined number of retained editing events in the two mating types, rather than the total number of potentially editable adenosines. Pairwise comparisons of these proportions among the three species were performed using z-ratios, and the *p*-values were adjusted using Tukey’s method for a family of three estimates.

### 2.7. Codon Usage and Structural Context Analysis

To determine whether synonymous RNA editing events shift codons toward more or less frequently used synonyms, a genome-wide codon usage table was constructed for each species from the full set of annotated coding sequences, and Relative Synonymous Codon Usage (RSCU) was calculated for each codon within its amino acid’s synonymous codon family. For each synonymous editing site, the reference and edited codon were determined directly from the transcript coding sequence, and each site was classified according to whether the edited codon had a higher or lower RSCU value than the reference codon. Every extracted codon change was cross-validated against SnpEff’s independently generated amino-acid annotation for the same site to confirm correct coding-frame assignment. For nonsynonymous editing sites detected independently in two or more lifecycle stages, the position of the affected residue was compared against each protein’s predicted domain, transmembrane topology, and intrinsic disorder annotations (Pfam, PROSITE, PANTHER, Gene3D, SUPERFAMILY, Phobius, TMHMM, MobiDB-lite), retrieved via the Ensembl REST API, to assess whether recurrently edited residues localize to structurally conserved or catalytic regions versus flexible or peripheral regions of the protein [37,38,39,40,41,42,43,44,45].

## 3. Results

### 3.1. A Low Number of A-to-I RNA Editing Sites Were Detected in MI

**Figure 3:**
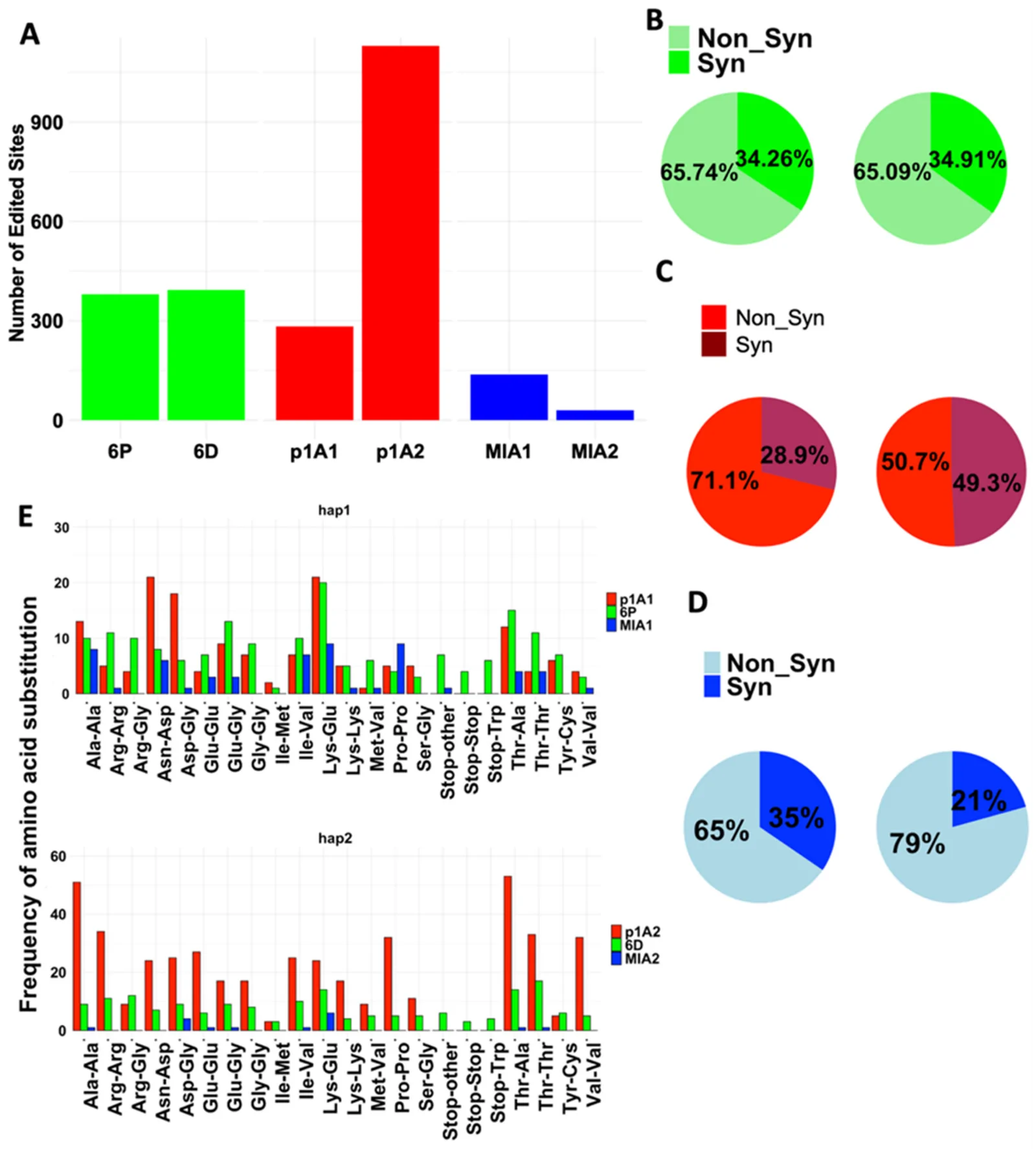
(**A**) Comparison of the number of editing sites in haploid strains of three species. (**B**–**D**) represent synonymous vs. nonsynonymous codon changes: Panel (**B**) represents MvSup haploids 6P (left) vs. 6D (right). Light green color represents nonsynonymous codon changes, and green color represents synonymous codon changes. Panel (**C**) represents MVLG strains p1A1 (left) vs. p1A2 (right). Red color represents nonsynonymous codon changes, and maroon color represents synonymous codon changes. Panel (**D**) represents MIA1 (left) vs. MIA2 (right). Light blue color represents nonsynonymous codon changes and dark blue color represents synonymous codon changes. Panel (**E**) represents frequency measured as the number of occurrences of different types of codon changes out of the total number of RNA edits in both mating types of all three species. In this figure, hap1 represents a1 mating types, and hap2 represents a2 mating types. Thr to Ala, Lys to Glu, and Ile to Val substitutions were observed across both mating-type groups of all three species; Asn toAsp was additionally common among the a1 strains, whereas Asp to Gly was observed among the a2 strains.

We have developed a pipeline to identify and analyze the RNA editing sites in mRNA of MvSup, MI, and MVLG, each requiring its own specific host species to complete its lifecycle. The data were analyzed for three different stages of the lifecycle of these fungi. After accounting for SNPs among mating-type strains for each species, MVLG p1A2 haploid strains had more editing sites than MVLG p1A1 and all other haploid strains of the two species MvSup and MI. Also, MI haploid strains had fewer edited sites than all other haploid strains of MvSup and MVLG. In fact, among all three species of *Microbotryum*, the a2 haploid strain of MI displayed the lowest number of editing sites (Figure 3A).

Within each species, the proportion of A-to-I RNA edits differed between haploid types. To ensure that differences were not due to sequencing errors or to SNPs between strains used and the respective reference genome, the genomes of each strain used in this study were re-sequenced in duplicate. SNPs between these and the respective reference genome were noted; then SNPs between replicates of the same strain were identified and removed from the catalog of predicted RNA edits for that strain. The variation between replicates was the differences observed between two replicates of each mating type. For MI a1 haploids, 772 SNPs were identified, while the MI a2 haploids had 692 SNPs. In the case of p1A1, the comparison between replicate 1 and replicate 2 revealed 2093 SNPs, whereas p1A2 showed 1068 SNPs. For 6D, the difference between replicate 1 and replicate 2 was 737 SNPs, and for 6P, it was 700 SNPs. After accounting for SNPs between replicates, the differences in genomic DNA between the a1 and a2 haploids for each species were significant. In MI, there are 4905 differences between the two mating types, with most SNPs located in regions specific to the MAT chromosome and minimal variation in other areas. Conversely, in MVLG, there are 3488 SNPs distinguishing the two mating types, with notable differences also found outside the MAT chromosome. For Mvsup, there were 6012 SNPs observed between the two mating types. In MvSup, 6P had a similar proportion of edits to 6D (6P proportion of edits = 0.4915, CI: 0.456–0.527). In MVLG, the p1A1 had a significantly lower proportion of edits than p1A2 (0.2147, CI:0.192–0.238). For MI, MIA1 had a significantly higher proportion of edits than MIA2 (0.8214, CI:0.762–0.880). Additionally, all three species differed from each other in the proportion of edits in haploid types (Supplementary Table S3). These differences between mating-type strains used for each species allowed us to ignore such SNPs when comparing the respective strains for RNA editing differences that could be considered mating-type specific. After quality filtering, candidate editing sites numbered 316, 44, and 387 for MI a1, a2, and mated samples, respectively; DNA-SNP control filtering reduced these to 185, 40, and 263 final editing sites. In MVLG, candidate counts were 595, 1218, and 2023 for p1A1, p1A2, and mated samples, narrowing to 283, 1045, and 1660 final sites after DNA-control filtering. In MvSup, candidate counts were 2727, 2195, and 2351 for 6P, 6D, and mated samples, reduced to 381, 394, and 256 final editing sites.

### 3.2. Three Types of Amino Acid Substitution Are Prevalent in the Haploids of Microbotryum spp.

When examining RNA editing occurrence, nonsynonymous changes were more frequent in all species and both haploid types (all differences in nonsynonymous and synonymous changes were significantly different from 0), except in MVLG p1A2, which had equal numbers of nonsynonymous and synonymous changes (estimated mean difference in nonsynonymous and synonymous changes: −0.0492, CI overlapped 0: −0.161–0.0631). In the MvSup strain 6P, 133 and 248 genes were synonymously and nonsynonymously edited, respectively, and in 6D, 135 and 259 genes were synonymously and nonsynonymously edited, respectively (Figure 3B). In MVLG p1A1, 82 and 201 genes were found synonymously and nonsynonymously edited, respectively, while in p1A2, 515 and 530 genes were found synonymously and nonsynonymously edited, respectively (Figure 3C). In MIA1, 64 and 121 genes were synonymously and nonsynonymously edited, respectively, and in MIA2, 8 and 31 genes were synonymously and nonsynonymously edited, respectively (Figure 3D).

When we compared the predicted amino acid substitutions of each haploid strain of all three species, we found mainly four types of substitutions common to all three a1 mating-type strains (p1A1, 6P, and MIA1): threonine (Thr ACG, ACC) to alanine (Ala GCG, GCC), asparagine (Asn AAG) to aspartic acid (Asp GAC), lysine (Lys AAG) to glutamic acid (Glu GAG) and isoleucine (Ile ATT, ATC) to valine (Val GTT, GTC). Interestingly, three types of substitutions, Thr to Ala, Lys to Glu, and Ile to Val, were also prevalent in the a2 mating-type haploid strains of all three species. In addition, Asp (GAC, GAT) to Gly (GGU, GGC) substitutions were also prevalent in all three a2 mating-type strains (p1A2, 6D, and MIA2). Moreover, synonymous and nonsynonymous codon changes occurred in STOP codons of both mating types of MvSup. We observed STOP (UGA) to Trp (UGG) codon conversion in 6P and 6D. Synonymous codon changes also occurred in both mating types for Ala (GCA>GCG), Thr (ACA>ACG), Pro (CCA>CCG), Glu (GAA>GAG), Val (GUA>GUG) (Figure 3E). An analysis of the preferred codons for MVLG revealed that for the most degenerate position (i.e., third position), seventeen out of eighteen had a G or C base [19]. Therefore, synonymous codon changes primarily influence the GC composition of the edited genes. That suggests RNA editing is essential for preferred codon selection based on GC composition in *Microbotryum* spp. Consistent with this GC-composition bias, a genome-wide analysis of codon usage showed that synonymous editing events shifted codons toward the more frequently used (optimal) synonymous codon in 94–100% of sites in MI and 92–94% of sites in MVLG across all haploid and mated conditions examined, with the same directional bias observed in an independently validated subset of MvSup sites (92–98%).

### 3.3. Analysis of Unique RNA Editing Sites in Haploids and Mating Stage of Life Cycle

**Figure 4:**
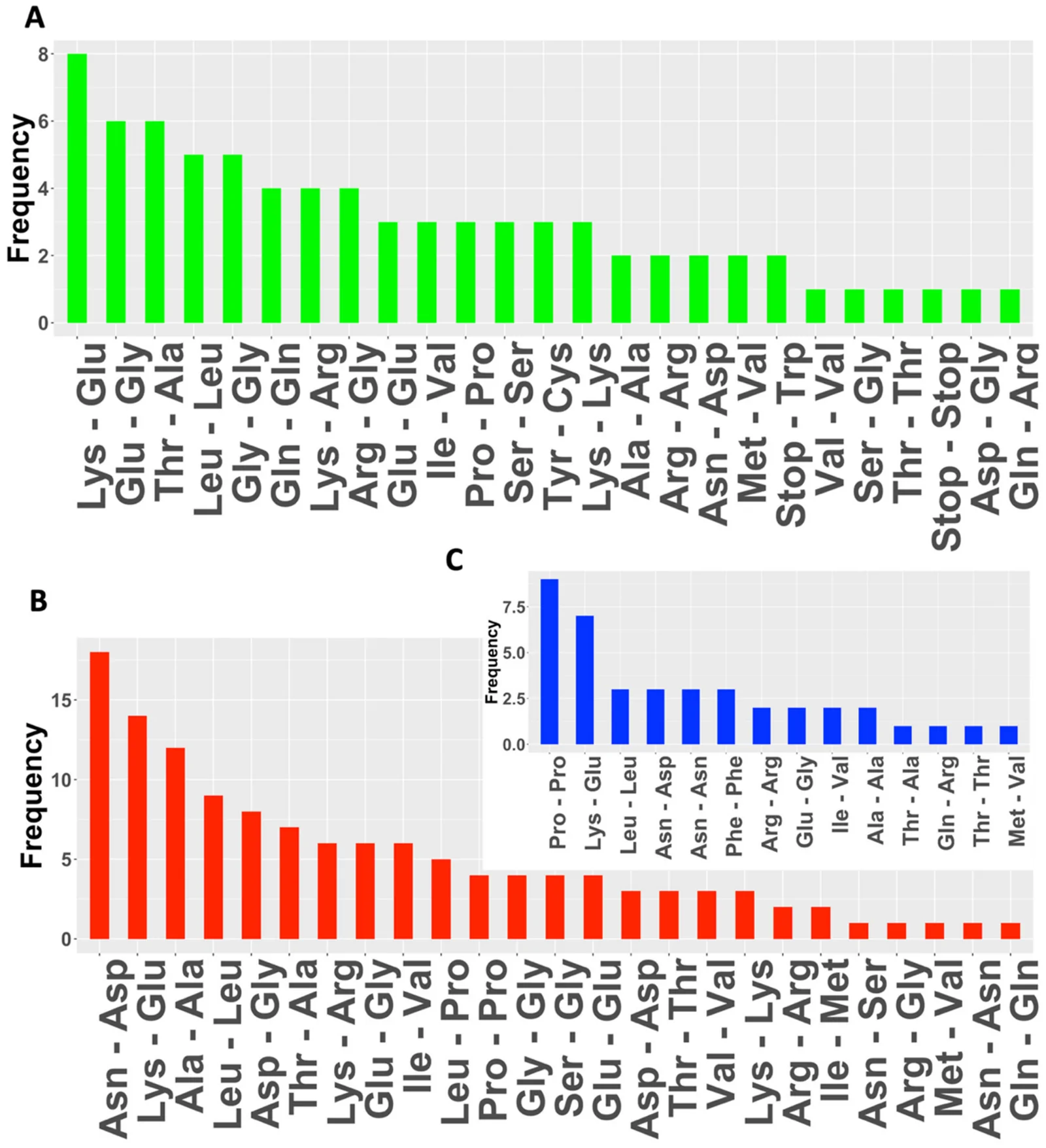
Amino-acid substitutions associated with unique nonsynonymous RNA editing sites in the a1 haploid mating types of three *Microbotryum* species. (**A**) MvSup-6P (green; n = 71 unique nonsynonymous RNA editing sites); (**B**) MVLG-p1A1 (red; n = 120 unique nonsynonymous RNA editing sites); and (**C**) MIA1 (blue; n = 43 unique nonsynonymous RNA editing sites). Amino-acid labels indicate the predicted change from the original amino acid to the amino acid resulting from the edited codon. The *y*-axis (“Frequency”) represents the number of occurrences of each amino-acid change. Among the nonsynonymous changes, Lys to Glu was one of the most frequent substitutions across the three a1 haploids, while Asn to Asp was the most frequent substitution in MVLG-p1A1; alanine was also a shared preferred substitution outcome in MvSup-6P and MVLG-p1A1.

**Figure 5:**
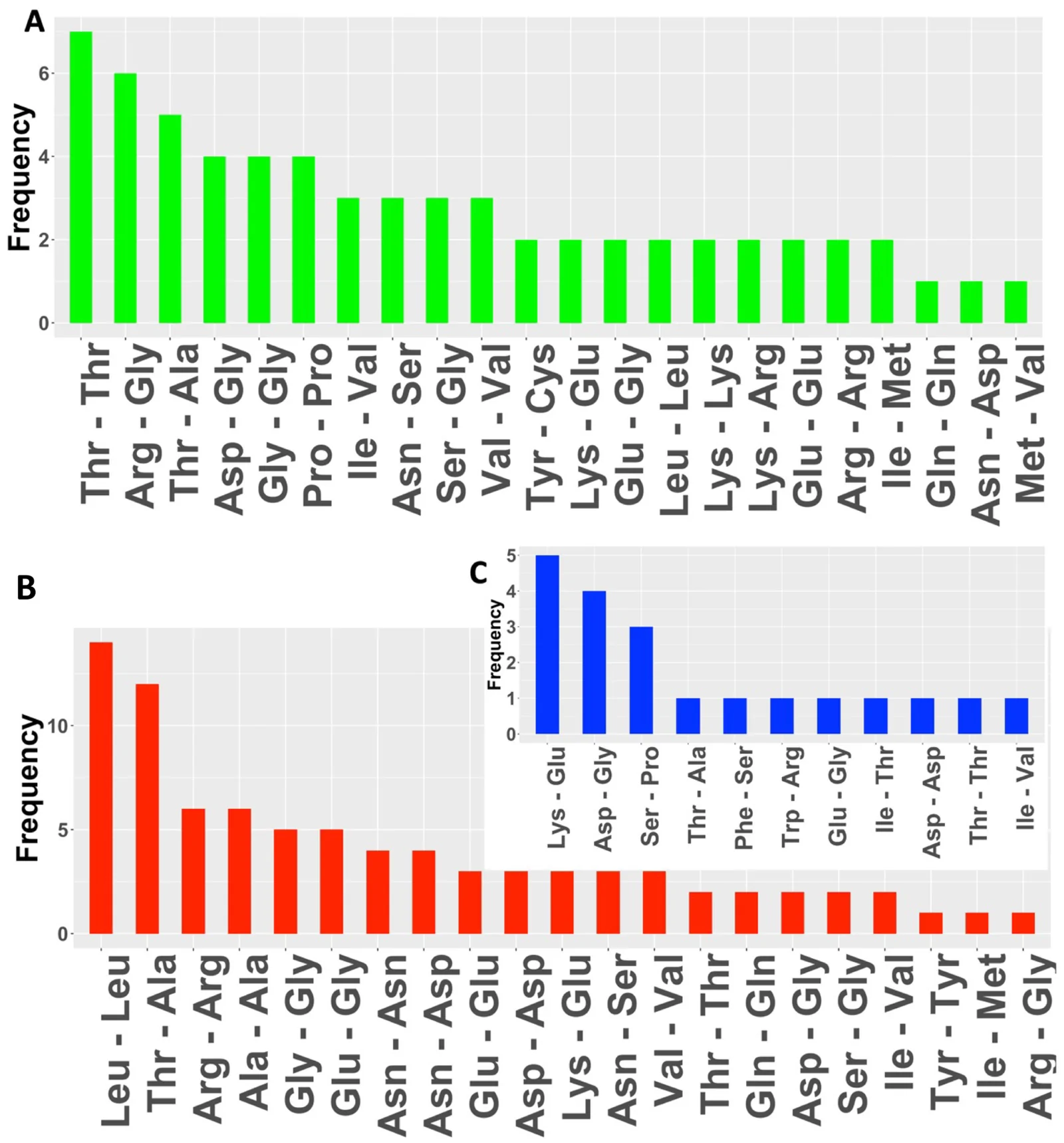
Amino-acid substitutions in genes uniquely edited in the a2 haploid mating types of three Microbotryum species. (**A**) MvSup-6D (green; n = 91 unique nonsynonymous RNA editing sites); (**B**) MVLG-p1A2 (red; n = 116 unique nonsynonymous RNA editing sites); and (**C**) MIA2 (blue; n = 22 unique nonsynonymous RNA editing sites). Amino-acid labels indicate the predicted substitution from the original amino acid to the amino acid resulting from the edited codon. The *y*-axis (“Frequency”) represents the number of occurrences of each amino-acid substitution shown on the *x*-axis. Among these uniquely edited sites, Thr to Ala was a prominent substitution in MvSup-6D and MVLG-p1A2, whereas glycine-producing substitutions were also prominent in MvSup-6D and MIA2; Lys to Glu was the most frequent substitution observed in MIA2.

Among all the RNA editing sites we identified for MvSup, 71 and 91 unique sites were edited nonsynonymously in a1 and a2 mating types, respectively. Likewise, for MVLG, 120 sites were edited nonsynonymously that are unique for the a1 mating type, and 116 unique sites were edited nonsynonymously in the a2 mating type. Similarly, in MI, 43 and 22 unique sites, respectively, were edited nonsynonymously in the a1 and a2 mating types. When we compared the amino acid substitutions, we found that in both haploids of MvSup and MVLG, Ala (GCG, GCC) was one of the shared preferred amino acid substitutions after nonsynonymous codon changes for both species (Figure 4A,B and Figure 5A,B). However, in the a1 mating type of MvSup, MVLG, and both haploids of MI, Glu (GAG) was another preferred codon for nonsynonymous codon changes. In M1A2 and both mating types of MvSup, Gly (GGA, GGU) was the other preferred codon for nonsynonymous codon changes (Figure 4C and Figure 5C). Furthermore, to confirm the editing sites that are exclusive to one haploid but absent in the other mating type of the same species, we used PCR to verify whether these potential editing sites, unique to one strain, are truly present when the genomic DNA is the same in both strains (Supplementary Figure S1). Most validated sites showed complete editing (100%); however, one site in *Microbotryum superbum* (*M. superbum* tig00000084: 3571980; Figure S1D) showed partial editing, with approximately 70% of reads supporting the edited base, suggesting that editing efficiency may vary among loci.

### 3.4. Important Genes Edited Uniquely at the Mating Stage of MvSup

**Figure 6:**
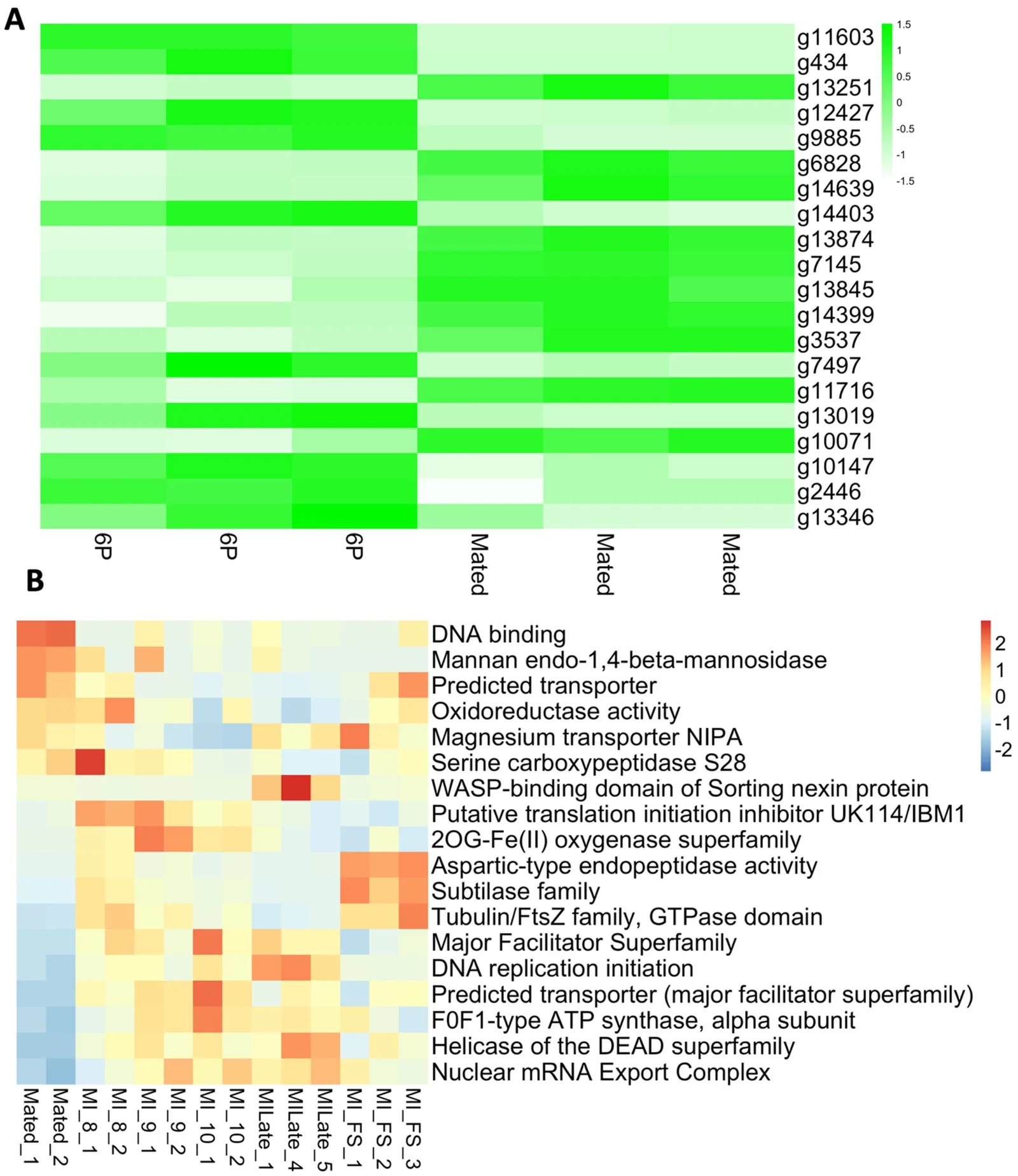
(**A**) Represents unique genes for MvSup at the mated stage that were either up- or downregulated in the haploid stage. Shade of green indicates degree of up- or downregulation; (**B**) Unique genes for MVLG at the mated stage that were either up- or downregulated in the infection stage, based on color scale inset. *x*-axis represents samples collected at the mated stage (first two samples from left) and at different stages of infection (last 12 samples on the right).

In the mating stage of MvSup, we found that a synonymous codon change occurred in a mitogen-activated protein kinase domain-containing protein at codon position 25 for Gly within the conserved region of the protein (Supplementary Figure S2). Our analysis of gene expression data indicated that certain genes, which were uniquely edited during the mating stage of MvSup genes, show a tendency to be upregulated in the haploid stage but downregulated during the mating stage, and vice versa. To assess the statistical significance of these differences, expression values for the haploid (6P) and mated stages (three biological replicates each) were compared for the 20 genes shown in Figure 6A using a two-sample Welch’s *t*-test per gene, with the Benjamini–Hochberg procedure applied to control the false discovery rate (FDR) across the gene set. Seventeen of the 20 genes were significantly differentially expressed (FDR < 0.05), and all 20 showed at least a two-fold change in expression (|log2 fold change| > 1) between stages. For instance, a gene (g6828) that codes for a protein involved in the phytosteroid metabolic process was upregulated only during the mating stage. Another gene (g3537) that codes for a protein involved in the regulation of the apoptotic process was also upregulated during the mating stage when compared with the haploid conditions (g6828: log2FC = 1.47, FDR = 0.004; g3537: log2FC = 1.36, FDR = 0.018) (Figure 6A and Supplementary Table S4).

### 3.5. RNA Editing of Subtilisin-like Proteases Was Found Only at the Mating Stage of MVLG, but Not in Other Stages of the Lifecycle

Thirty-two genes that are unique for MVLG at the mated stage were either up- or downregulated in the infection stage. Differential expression between the mated stage and each infection substage (MI_8, MI_9, MI_10, MILate, MI_FS) was assessed using edgeR; genes were considered significant at FDR < 0.05. All 31 genes listed in Table 1 were significantly differentially expressed in at least one such comparison. For example, one gene associated with serine-type endopeptidase/Subtilase activity was downregulated in the mating stage. RNA editing of that gene at the mating stage caused codon changes from Thr (ACG) to Ala (GCG) at position 681. The role of subtilisins in plant pathogenic fungi has been highlighted, suggesting their important role in plant infection [46,47]. Also, we observed that most genes related to transport were downregulated during the mating stage when compared with the infection stage; one exception, a gene encoding a predicted transporter of the major facilitator superfamily (MVLG_05629), showed the opposite pattern, with higher expression during mating and lower expression during infection (log2FC = −3.87, FDR = 2.6 × 10^−31^, MILate vs. Mated) (Table 1). Moreover, a gene associated with DNA binding was strongly upregulated in the mated stage and generally had no change in expression in the infection stage (Figure 6B, Table 1).

### 3.6. Common Genes Edited in Haploids and Mating Stages of MI

**Figure 7:**
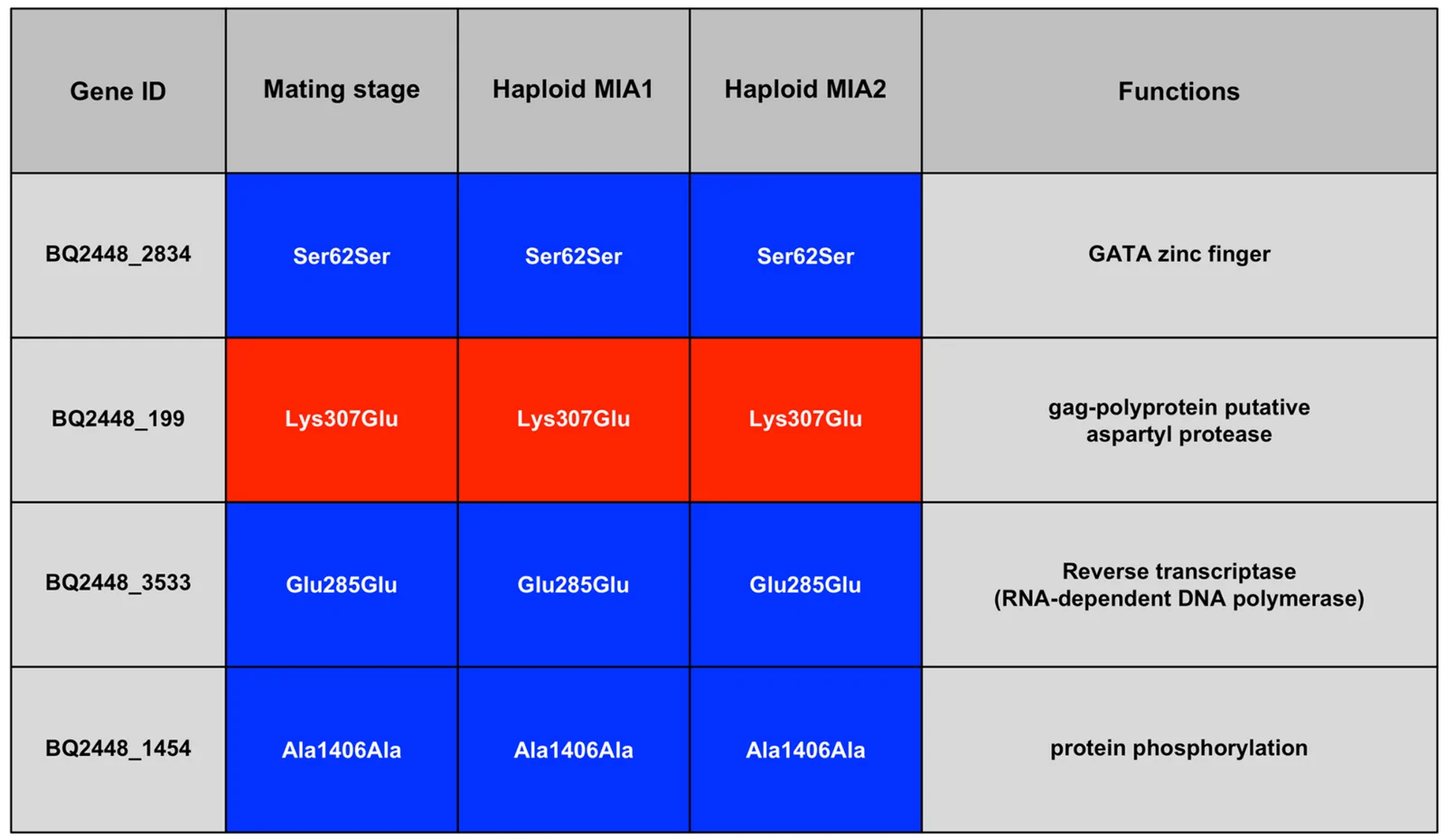
Synonymous vs. nonsynonymous codon changes occurred in the genes edited in MIA1, MIA2, and the mated strains of MI. Blue represents synonymous codon changes. Red represents nonsynonymous codon changes. This figure represents functions of all the genes that are commonly edited in two haploid stages and the mating stage of MI.

We observed very few editing sites in common for the genes of MIA1, MIA2, and mated stages (Figure 7). Of these four common genes, some had only nonsynonymous changes across all stages, and some had only synonymous changes across all stages. For example, nonsynonymous editing was observed with the genes associated with the gag-polyprotein putative aspartyl protease, and synonymous changes were observed with the genes associated with protein phosphorylation activity in all stages of MI.

### 3.7. Unique Genes Edited in Haploids and Mating Stages of MI

In these analyses, we identified genes that were edited only at the mating stage. Interestingly, two proteins, according to Gene Ontology (GO) annotations, play a pivotal role in vesicle-mediated transport (GO:0016192) and aspartic-type endopeptidase activity (GO:0004190) (Table 2). It has been suggested that endosomal and extracellular vesicle-mediated transport mechanisms are involved in the secretion of small RNAs at the fungal-plant interface [48].

### 3.8. Analysis of Shared Gene Functions Across Life Stages in MvSup and MVLG

**Figure 8:**
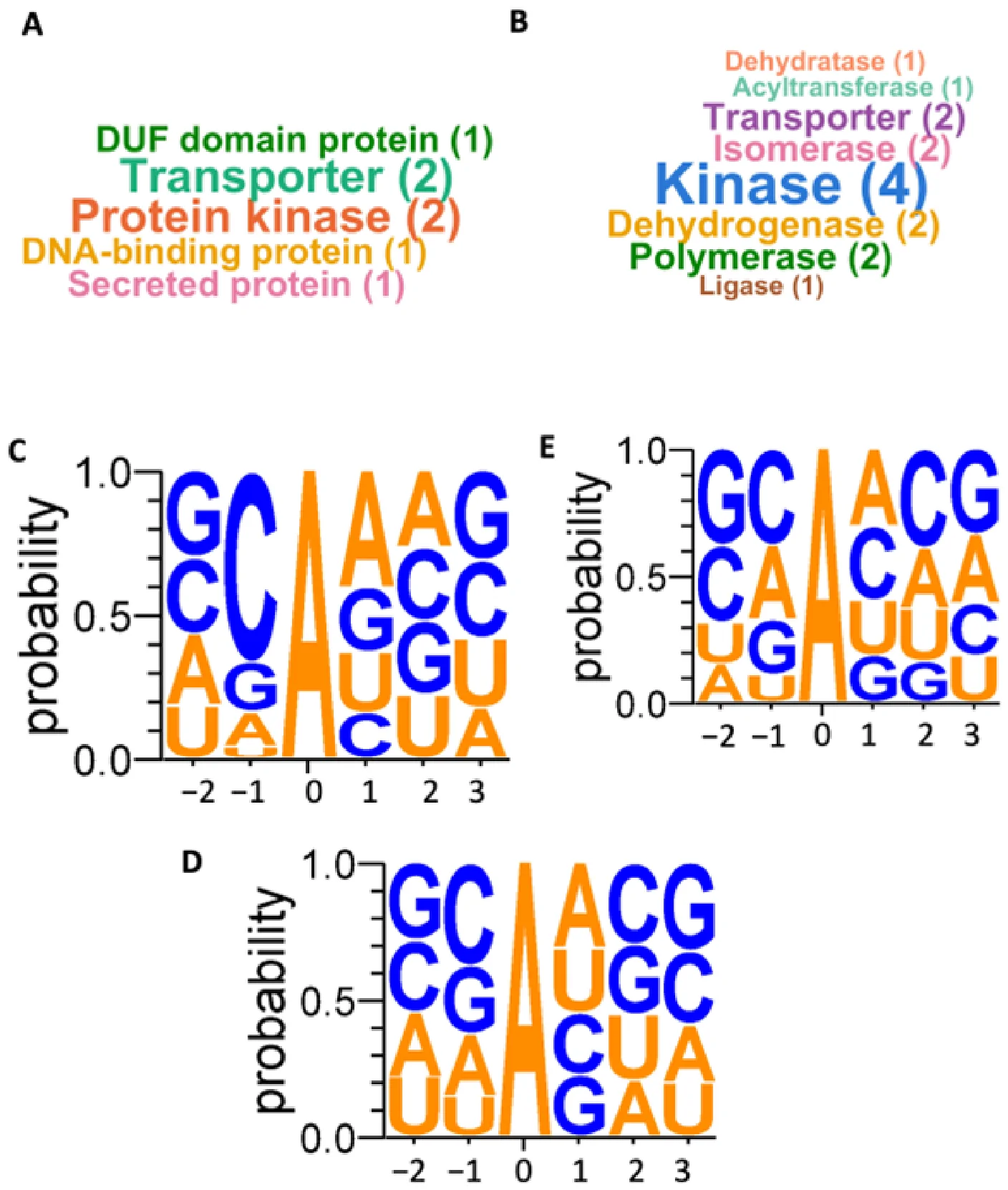
(**A**) Word cloud plot of the function of the genes that were edited during haploids, mating, and infection stages of MVLG. (**B**) Word cloud plot of the function of the genes that were edited during haploids, mating, and infection stages of MvSup. Word size is proportional to the frequency of each functional category, and the number in parentheses indicates the number of genes assigned to that category. Transporter and protein kinase were the most represented categories. (**C**–**E**) represent the identification of the preferred motif for RNA editing during the mating stage of MvSup, MVLG, and MI, respectively.

**Figure 9:**
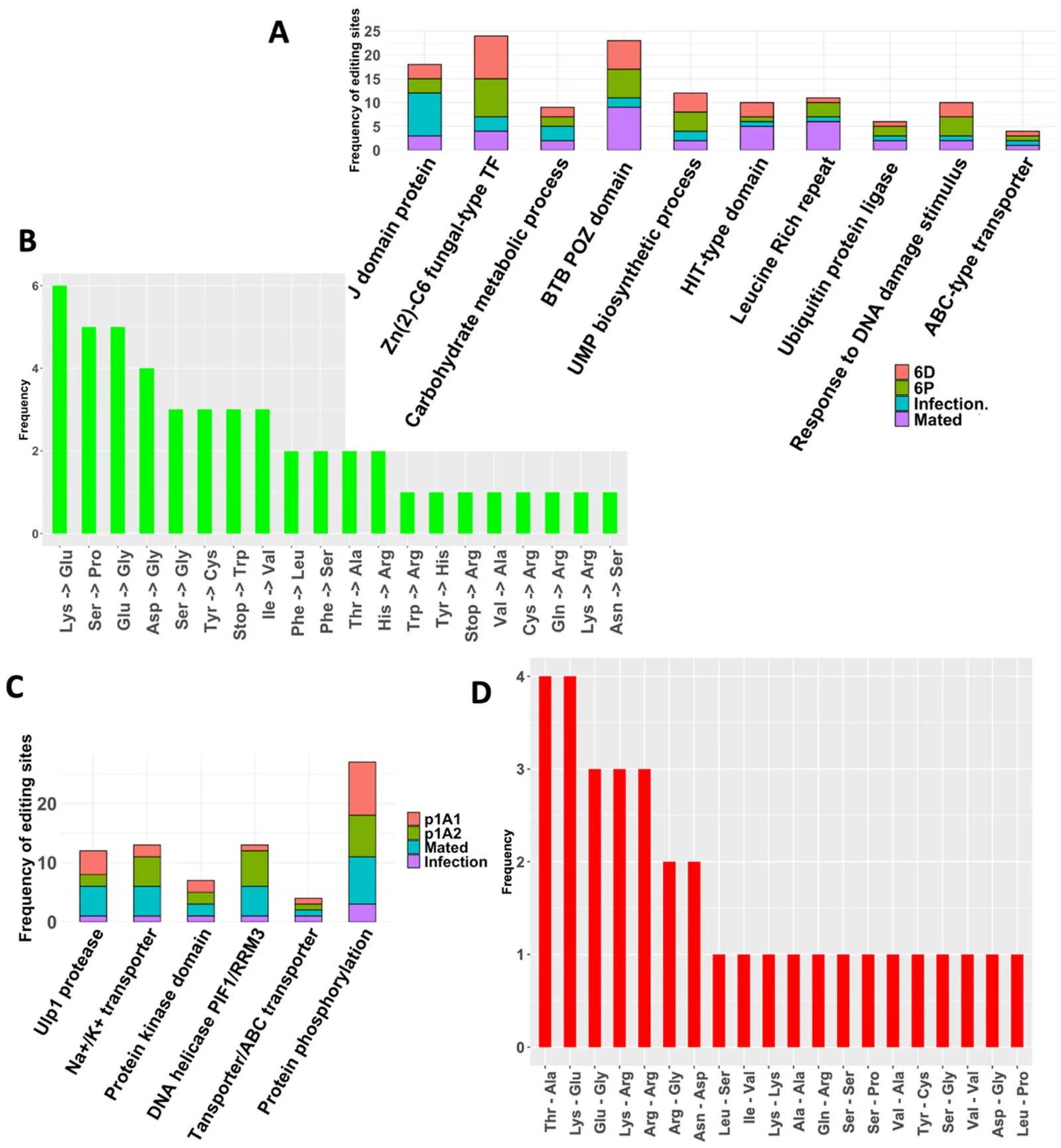
(**A**,**C**) Frequency of editing sites was compared between the common genes that are edited in all three stages (haploid, mating, and infection stages) of the lifecycle of MvSup and MVLG, respectively. (**B**,**D**) represent the distribution of amino substitutions in genes that are common for all three stages (haploid, mating, and infection stages) of MvSup and MVLG, respectively. For panels (**A**,**C**), frequency represents the number of RNA editing sites for each gene shown on the *x*-axis. For panels (**B**,**D**), frequency represents the number of occurrences of each amino-acid substitution shown on the *x*-axis. Lys to Glu was the most frequent amino-acid substitution in MvSup, whereas Thr to Ala was the most frequent substitution in MVLG. Among the commonly edited genes, the J-domain-containing protein showed more editing sites during infection in MvSup, whereas the Na^+^/K^+^ transporter showed the highest editing frequency during mating in MVLG.

Where RNA editing was found in all stages of the fungal lifecycle of MvSup and MVLG, genes associated with transport and kinase activity were represented in both species (Figure 8A,B). Distribution of amino acid substitutions in all the common genes edited during haploid, mating, and infection stages of MvSup and MVLG can provide insights into the evolutionary history of *Microbotryum*. We can infer evolutionary relationships, adaptation processes, and the conservation of specific amino acids in critical protein regions by comparing the substitutions between these species. In the mating phase of three species, we wanted to determine which motif was favored by the responsible enzyme(s). We found that during the *Microbotryum* mating stage, there was a preference for cytosine (C) at the −1 position (Figure 8C–E). We observed that within the common genes, for those edited in all stages of MVLG, Thr (ACG, ACC) to Ala (GCG, GCC) substitution was the most preferred, while Lys (AAG) to Glu (GAG) was preferred for MvSup (Figure 9B,C).

We identified that among all the genes where RNA editing occurred in every stage of the fungal life cycle in MvSup, five genes had more editing sites than those found for other genes edited in common for all stages of the lifecycle for that species. These five genes code for: J domain-containing protein, BTB POZ domain-containing protein, Leucine Rich repeat domain, carbohydrate metabolic process, and Zn (2)-C6 fungal-type transcription factor. Among the common genes, the gene encoding the J domain-containing protein exhibited a greater number of editing sites during the infection stage compared to all other stages. Also, three genes had more editing sites in the mated stage compared to all other stages: these genes code for the protein containing a BTB POZ domain, a Leucine Rich repeat domain, and a protein containing a HIT-type domain (Figure 9A).

In MVLG, a gene that encodes a Ulp1 protease, a member of the ubiquitin-like protease 1 (Ulp1) cysteine protease family, had more editing sites in the mated stage compared with other stages. This protein plays a crucial role in fungal mating and is responsible for the maturation of SUMO and the processing of the precursor of the ubiquitin-like Smt3 protein into its mature form, as well as the cleavage of Smt3 from its conjugates [49,50].

### 3.9. Transport-Related Genes: A Shared Editing Profile Between MvSup and MVLG

Genes associated with transport were the most commonly edited across all lifecycle stages examined for both MVLG and MvSup. Of particular note, a gene that codes for an ABC-type transporter was edited in all stages in both species (Figure 9A,C). ABC-type transporters are a crucial component of drug resistance and detoxification mechanisms in fungi. These transporters, which belong to the ATP-binding cassette (ABC) superfamily, are involved in efflux-mediated resistance to antifungal agents [51,52]. Moreover, a gene that codes for a Na^+^/K^+^ transporter protein had the most editing sites during the mating stage compared to other stages in MVLG (Figure 9C).

### 3.10. Comparison of Late-Stage Male-Infected Flowers (MILate) vs. Late-Stage Female-Infected Flowers (FILate) in MVLG

The preferred host for infection by *Microbotryum* species is a male plant, since the fungus replaces pollen in the anthers of its host. When the host species is dioecious, i.e., where the male and female reproductive parts are found in separate plants, the fungus causes the ovary to abort, and a pseudo-anther is produced, allowing completion of the fungal lifecycle in a female plant. Typically, the degree of infection of such pseudo-anthers is less than for an infected male flower. Thus, we were interested in seeing if there were differences in RNA editing during the respective host infections. There were 533 edited sites observed in MILate of MVLG and 259 edited sites in FILate. Other than 97 shared genes, 28 and 26 genes were unique in the MILate stage and FILate stage, respectively. A gene encoding a protein with a lytic transglycolase domain (PF03330) was edited only in the FILate stage. The lytic transglycolase plays a crucial role in the infection process of fungi in plants [53,54,55]. Another gene encoding peptidyl-prolyl cis-trans isomerase was also edited only in the FILate stage. Additionally, we also observed in the MILate stage that a unique edited gene was that encoding methylenomycin: H^+^ antiporter, a protein found to play a significant role in the salt tolerance and infection process of fungi in plants [55,56,57] (Supplementary Table S5).

**Figure 10:**
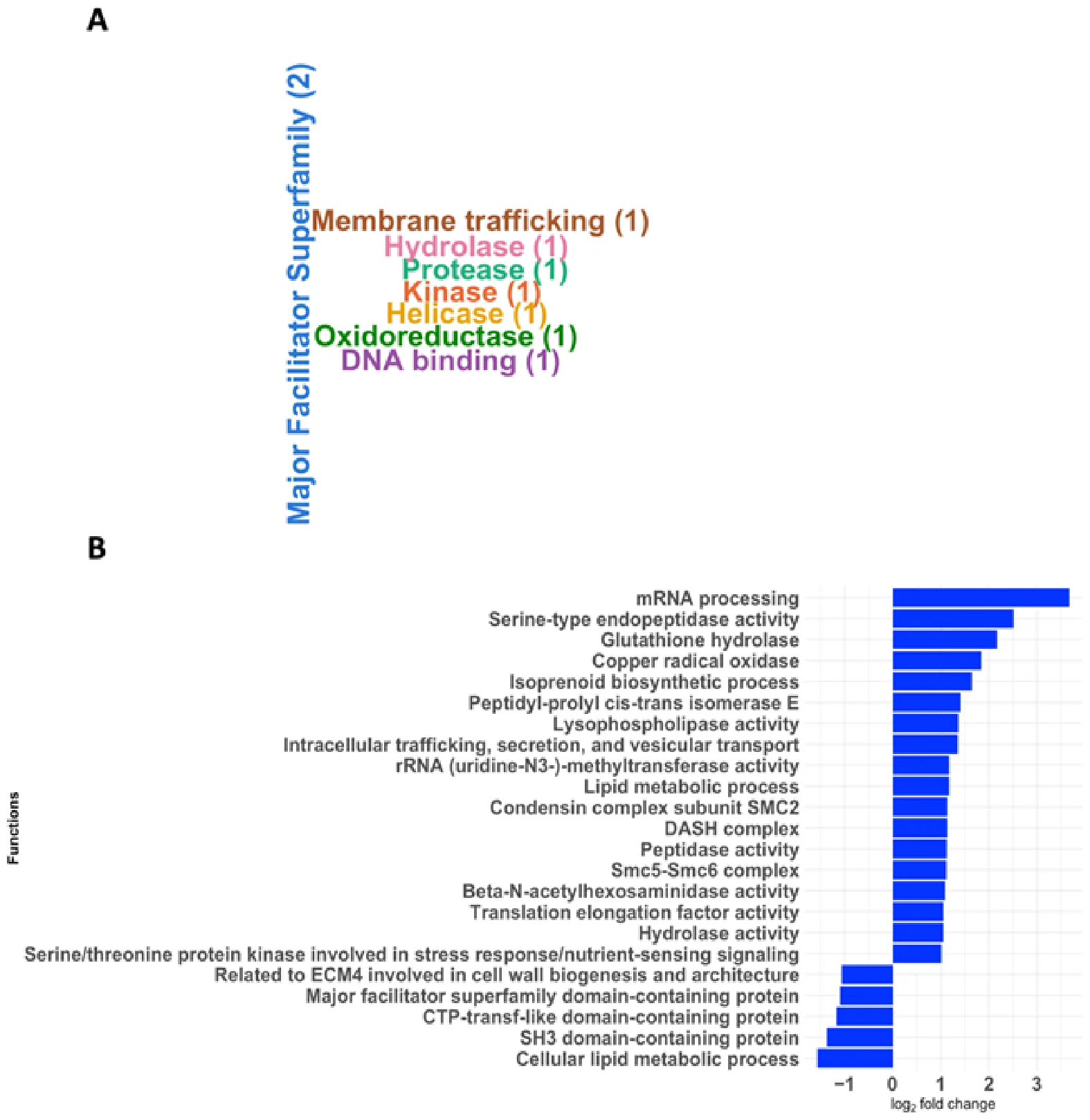
(**A**) Word cloud plot of the function of the genes edited only during the MILate infection stage in MVLG. Word size, identical in these examples, is proportional to the frequency of each functional category, and the number in parentheses indicates the number of genes assigned to that category. Among the functionally annotated genes shown (n = 9), the major facilitator superfamily was the most represented category. (**B**) Differential expression level (mated vs. infection stage) of the genes uniquely edited during the infection stage in MvSup.

An additional question was whether genes undergoing RNA editing were differentially expressed at the late infection stage in male flowers compared to the haploid stage of MVLG. We observed that among the two genes associated with the major facilitator superfamily (MVLG_05383 and MVLG_01436), MVLG_05383 was upregulated in the late infection stage in male flowers and the other was upregulated in haploids. The genes coding for flavin-binding monooxygenase and for CDC24, which has a PB1 domain involved in cytoplasmic signaling, were upregulated in the haploid stage compared to the infection stage (Supplementary Figure S3, Supplementary Table S6). Furthermore, the MILate stage-specific genes where RNA editing sites were identified encoded helicase, endopeptidase, and major facilitators (Figure 10A).

### 3.11. Uniquely Edited Genes During Infection Stage of MvSup

Differential gene expression between mated and infection conditions was examined for RNA-edited genes of MvSup that occurred only during the infection stage. We identified upregulated genes associated with functions like Isoprenoid biosynthetic process, Probable SCH9—serine/threonine protein kinase involved in stress response and nutrient-sensing signaling pathway, and Intracellular trafficking, secretion, and vesicular transport. At the same time, ECM4-like protein, which likely contains subtilisin-like serine proteases and an SH3 domain, was downregulated during infection conditions (Figure 10B).

## 4. Discussion

In the current study, phytopathogenic basidiomycete species of the *Microbotryum* fungal complex were examined for RNA editing at haploid, mating, and infection stages of their respective lifecycles. While there have been several previously published reports suggesting RNA editing in Basidiomycetes (including *Ganoderma lucidum* [58] and five species of Polyporales [59]), these reports have since been challenged. In part, this was due to the fact that not only A-to-I changes but also A-to-G, G-to-A, C-to-U, and U-to-C alterations in transcripts were reported in these previous studies. Further investigation showed that RNA editing sites in the five Polyporales species were artifacts resulting from variations between the genomes of different isolates used for DNA and RNA extraction or simply from genome misassemblies [59]. Due to these issues, reliable demonstrations of RNA editing in Basidiomycetes have been lacking. Thus, we sought here to examine RNA editing in *Microbotryum*, while avoiding these pitfalls and to allow some comparisons with the few filamentous ascomycete species where RNA editing has predominantly been characterized in fungi, e.g., *F. graminearum*, *F. verticillioides*, *N. crassa*, *Sordaria macrospora*, and *Pyronema confluens* [1,3,4,60]. In addition, while other types of RNA editing (e.g., C-to-U) have been reported in humans [61], here we focused on editing involving only A-to-I changes, as these have been most studied so far in fungi. Also, inspection of the genomes of the three *Microbotryum* species in this study revealed multiple genes predicted to encode adenosine deaminases, some of which appeared more similar to ADARs found in animal species, while there was at least one putative ADAT-like enzyme encoded in each species. Interestingly, for at least MvSup and MVLG, the predicted ADAR-like homologues were upregulated during infection of their respective plant hosts (Supplementary Table S1).

We performed A-to-I RNA editing analysis, using our in-house pipeline (Figure 2), which includes several functions from the GATK workflow. To be included in these analyses, sites had to be found to have been edited at a minimum of 10% of the RNAs examined for a particular gene. In practice, for a given gene, the level of RNA editing ranged from a low of 46% up to 100% and, in some cases, the level of editing coincided with upregulation of the gene at a particular stage of the lifecycle. Some examples of genes that were upregulated in a particular stage and were also found to have a higher level of RNA editing included ones encoding FAD-binding domain-containing protein (MVLG_05396), CDC24 Calponin (MVLG_05912), and Helicase domain-containing protein (MVLG_07154). These genes were edited during both the haploid and infection stages of MVLG. However, when we analyzed gene expression, we found that these genes were upregulated in the haploid stage relative to the infection stage (MVLG_05396: log2FC = 7.28, FDR = 5.4 × 10^−11^; MVLG_05912: log2FC = 2.23, FDR = 0.015; MVLG_07154: log2FC = 4.47, FDR = 0.0034). Interestingly, the editing level percentages were also higher in the haploid stage (89–100%, depending on the position in the target RNA for the gene) compared to the infection stage (Supplementary Table S7). A systematic, quantitative relationship between editing level and expression change across the full set of edited genes was not tested in this study and represents a direction for future work. Moreover, among the three species, MvSup, MI, and MVLG, our results suggest that the total number of edits and the proportion of edits in each haploid type differ between species. Genetic factors and environmental adaptations may influence this variability in RNA editing, highlighting the complexity of RNA editing regulation across different species. In two different mating types, a1 and a2, nonsynonymous and synonymous codon changes occurred, suggesting these codon changes may play a role in the functionality or stability of the proteins, possibly related to the necessity of environmental adaptation during a particular stage of development. Synonymous codon changes can happen because the species have some preferred codon choice or to preserve protein function, so A-to-I RNA editing would therefore be necessary for these *Microbotryum* species. Different levels of editing among the mating types for the three species examined here may reflect variations in their hosts, which can significantly influence pathogenicity. For instance, one *Microbotryum* species might infect a broad range of plant host species (e.g., MvSup), adapting to diverse environmental conditions. In contrast, other species might specialize on a single host species, thriving only in a narrow ecological niche.

When a codon change occurs leading to a Thr to Ala substitution in a protein, it has a significant impact on the phosphorylation processes. Thr is a common target for phosphorylation due to a hydroxyl group present in its side chain, providing a target for the addition of phosphate. In contrast, Ala lacks the hydroxyl group, so it would not be able to undergo phosphorylation [7,62]. This alteration can affect biological processes associated with phosphorylation and signaling pathways, which regulate various cellular functions such as cell cycle progression, gene expression, and signal transduction [63]. However, we have not directly tested whether editing at these positions alters phosphorylation status or protein function in this study. Confirming this would require direct evidence, such as phosphoproteomic analysis, targeted mutagenesis, or structural modeling of the edited residues, and represents an important direction for future work.

We further investigated whether the observed pattern of codon usage bias in synonymous editing (Section 3.2) corresponds to a shift toward more efficiently translated codons. Using a genome-wide codon usage table built from each species’ full transcript set, we found that synonymous A-to-I edits shift toward the more frequently used (optimal) synonymous codon in 94–100% of sites in MI and 92–94% of sites in MVLG, across all haploid and mated conditions examined, with a smaller independently validated subset of MvSup sites showing the same direction (92–98%). This aligns with the GC-composition preference mentioned earlier and shows that synonymous editing in these species does not add rare codons that slow translation and cause ribosomal pausing. Instead, editing moves codon usage toward faster, more efficient codons.

For nonsynonymous substitutions, we examined the position of recurrently edited residues (those detected independently in multiple lifecycle stages) relative to each protein’s known domain architecture, transmembrane topology, and predicted intrinsic disorder. In several cases, edited residues fell in flexible or peripheral regions rather than within core structural or catalytic elements: for example, all recurrently edited residues in a predicted MVLG cation transporter (Trk/HKT family) localized to its large non-cytoplasmic N-terminal domain, upstream of its transmembrane-spanning core, and several recurrently edited residues in other proteins overlapped predicted intrinsically disordered regions, which are inherently solvent-exposed. A smaller number of edits occurred closer to catalytic or structurally conserved elements, including a substitution adjacent to the conserved kinase catalytic loop and, in *M. intermedium*, two edits within the integrase domain of a retrotransposon-derived polyprotein. These observations suggest that most repeated nonsynonymous editing events probably do not greatly disrupt the overall protein structure. However, some may have direct functional effects that need more study, such as using protein structural modeling to examine specific edited residues.

In MvSup and MVLG, of the unique genes only edited in the haploid strains, Ala was the most common codon after nonsynonymous substitution. However, when we compare the three species, Lys to Glu conversion is the common amino acid substitution among all the mating types. According to some studies of plant species, most RNA editing events in these organisms result in nonsynonymous modifications [64]; more than 95% of these editing events alter the amino acid sequence, often increasing the hydrophobicity of the proteins in the chloroplasts of leaves across 21 plant species, including ferns, gymnosperms, and angiosperms. Protein folding and stability can be improved by this hydrophobicity, which may be essential for the organism’s capacity to respond to environmental stressors. The reasons behind this difference among the *Microbotryum* species in the current study could be genetic or environmental factors, which may influence the evolution or biological functions of these three species for better adaptation in the environment [65]. Furthermore, because of their sequence context and many adenosines, certain codons are more vulnerable to editing. For example, it has been discovered that the Lys codons, which feature two adenosines, are abundant in editing motifs, indicating a codon-dependent preference for editing. Because of this codon selectivity, specific amino acids, such as Ala, can be preferentially expressed through A-to-I editing, which can affect the interactions and functional characteristics of the protein [66].

In our research, we examined RNA editing from A-to-I during the sexual development phase by comparing *Microbotryum*, a genus of basidiomycete fungi, with ascomycetes such as *Neurospora crassa*. Our findings revealed that three distinct *Microbotryum* species also underwent editing throughout their respective life cycles. In the context of motif identification, another ascomycete, *Fusarium graminierum*, exhibited a preference for U at the −1 position, while *Microbotryum* showed a preference for C at the same position during the mating phase [67]. The patterns of RNA editing thus seem to vary between the ascomycetes examined previously and the *Microbotryum* species examined here, although broader taxonomic sampling is needed to determine the evolutionary significance of these differences.

The varying preferences for nucleotides at −1 positions within editing motifs might indicate differences in the RNA editing mechanisms or regulatory elements among these fungal groups. The variation in motif preferences between these ascomycetes and *Microbotryum* species during their mating may suggest differences in regulatory mechanisms that control RNA editing in these fungi.

In the following paragraphs, several specific protein examples are discussed where A-to-I RNA editing is predicted to alter protein function during different stages of the *Microbotryum* lifecycle. When comparing these with ascomycetes, e.g., *N. crassa*, RNA editing from A-to-I was studied during the sexual stage of development. Among the various genes that underwent editing throughout their life cycle, it was noted that the genes encoding the GATA-type zinc finger protein ASD4, DEAD/DEAH box RNA helicase, aspartic proteinase, serine protease, vesicle-mediated transporter Vid24, carboxypeptidase cpdS, ATP-binding cassette transporter, major facilitator superfamily transporter protein MFS1, and Ca^2+^/H^+^ antiporter were edited [5]. Genes with similar functions were also edited in *Microbotryum.* In MI, genes uniquely edited during the mating stage included genes annotated for vesicle-mediated transport and aspartic-type endopeptidase activity. Other genes with nonsynonymous editing changes were associated with proteolysis and cell redox homeostasis, including oxidoreductase and cysteine-type peptidase activities. Functions such as the activity of oxidoreductase and cysteine-type peptidase play a significant role in proteolytic pathways and redox reactions, which are crucial for cellular metabolism as well as defense from oxidative damage [47]. Additionally, in our study, we found that the three different species of *Microbotryum* also experienced editing during different stages of their life cycle. For instance, in MVLG, the gene associated with serine-type endopeptidase (MVLG_02763) was downregulated in the mating stage relative to infection (log2FC = 6.95, FDR = 2.8 × 10^−88^). RNA editing of that gene at the mating stage caused codon changes from Thr to Ala at position 681. In the context of subtilisin-like proteases in fungi, the substitution of a codon from Thr to Ala could potentially impact the enzymatic activity and substrate specificity of the protease [68,69]. This inference is based on precedent from the subtilisin literature rather than direct evidence from the present study. Subtilisin-like proteases play crucial roles in various biological processes, including plant defense, fungal virulence, and mutualistic interactions [70]. Therefore, changing the amino acid composition of these proteases in MVLG could affect the ability to cleave specific substrates, potentially affecting their biological functions. As there is a significant relationship between RNA editing and gene expression, this type of editing can significantly diversify gene expression and affect mRNA stability in other systems [71,72,73]; whether this applies specifically to the genes and editing sites identified in the present study was not directly tested. The significance of subtilisin-like proteases in fungal virulence has been emphasized, with studies reporting that these broadly distributed proteases are necessary for pathogenic fungi to be virulent [74]. Similarly, although ABC-type transporters are well established as mediators of drug resistance and detoxification in fungi, and a gene encoding an ABC-type transporter was found to be edited across all lifecycle stages examined in both MvSup and MVLG, whether the specific editing events identified in this gene alter its transport activity or contribute to drug resistance was not tested in this study; functional assays (e.g., efflux activity assays) or structural modeling of the edited residues would be needed to establish this directly. We also identified a synonymous codon change in a mitogen-activated protein kinase domain-containing protein, within a conserved region of the protein, during the mating stage of MvSup. As a synonymous substitution, this change does not alter the encoded amino acid; any functional consequence would need to act through mechanisms such as codon usage bias, translation kinetics, or mRNA processing, none of which were examined in this study. When we compared whether any genes edited in the *Microbotryum* mated stage were also edited in *Fusarium*, we did find some edited genes with similar functions: genes coding for ABC transporter, Heat shock factor protein, Histone-like transcription factor, Serine threonine-protein kinase, major facilitator superfamily MFS-1, Alpha beta hydrolase superfamily, F-box domain containing protein, and Vacuolar membrane protein.

A-to-I RNA editing occurred for the genes in common at each stage of the lifecycle of MVLG and MvSup, mostly those for transporter and transmembrane proteins. A-to-I RNA editing in RNAs of transporter proteins has yet to be extensively studied for fungi. However, transporter proteins are crucial in interactions between and among fungi and plants, particularly in mycorrhizal symbiosis [75]. Furthermore, identifying membrane proteins responsible for sensing and transporting biomass hydrolysates in anaerobic gut fungi highlights the importance of transport proteins in fungi [76]. A-to-I RNA editing in transporter proteins can be advantageous for fungi, possibly allowing them to colonize new environments and utilize previously inaccessible nutrient sources, making use of the modified forms of their transport proteins. We are interested in identifying the types of genes where RNA editing occurs in *F. graminearum* and *N. crassa* and comparing this inventory with those we observed in *Microbotryum*. Were these genes related to transporters or kinases, as we found, or do they belong to other categories? In *F. graminearum*, RNA editing is specifically observed in the AMD1 gene, which encodes a protein containing a major facilitator superfamily (MFS) domain. This gene plays a vital role in the maturation of asci and the release of ascospores during sexual development. The process of RNA editing involves A-to-I editing, which rectifies premature stop codons, allowing for the synthesis of complete and functional proteins [77].

In *N. crassa*, A-to-I RNA editing is regulated by developmental stages and predominantly takes place during sexual reproduction. This process leads to increased complexity in the proteome and has an impact on numerous genes associated with RNA silencing, DNA methylation, and histone modifications, including *sad-1*, *sms-3*, *qde-1*, and *dim-2*. Additionally, 50 pseudogenes that require A-to-I editing to produce complete proteins were discovered. These editing occurrences in *N. crassa* are generally advantageous and are subject to positive selection, thereby enhancing the organism’s adaptability [5].

RNA editing of the genes only occurring in the mated stage of the three *Microbotryum* species (e.g., associated with aspartic-type endopeptidase activity/serine-type endopeptidase activity/Calcium-dependent cysteine-type endopeptidase activity) is significant. Serine proteases in symbiotic fungi play important roles in both pathogenic and mutualistic associations that emphasize their importance in fungal biology and ecology [78]. These hydrolytic enzymes are essential for the breakdown of host tissues, which makes it easier for fungi to invade and get nutrients. Serine proteases, e.g., subtilisin-like proteases, have been demonstrated to break down the protective plant parasitic nematode cuticle in nematophagous fungi, enabling the fungi to successfully enter and infect their hosts [79,80].

Aspartic proteases are known for a variety of biological functions such as pathogenesis, nutrition, and biocontrol. They are a group of proteolytic enzymes that function in acidic environments and share the same catalytic domain [81]. These types of enzymes are involved in various processes including tissue invasion, migration, digestion, and reproduction in parasitic organisms [82]. For fungal infection, they may be involved in nutritional acquisition and play an important role in breaking down the tissue barriers and in destroying the molecules generated by the host plant as a defense [83]. Also, these types of enzymes have been identified as essential components in the biocontrol activities of fungi such as *Trichoderma harzianum*, where they play an important role among the enzymes released by the fungus [84]. Moreover, with respect to pathogenesis and nutrition, aspartic proteases have been shown to be involved in the dimorphism and pathogenesis of, e.g., *Ustilago maydis*, where vacuole proteases, including aspartic proteases, have essential functions in different physiological processes of the fungus [85]. Furthermore, aspartic proteases have been demonstrated to be involved in the thermo-dimorphism of *Paracoccidioides brasiliensis*, playing a crucial part in morphogenesis, cellular activity, immunology, and nutrition as well as the host invasion process [86]. In the context of plant-fungal interactions, cysteine proteases have been implicated in suppressing host immunity by inhibiting apoplastic cysteine proteases, allowing for biotrophic interactions of *U. maydis* with maize [87].

In comparison of MVLG MILate to the MVLG FILate stage, unique RNA editing was seen in the latter for a gene coding for a lytic transglycolase-like enzyme; these enzymes are responsible for the hydrolysis of glycosidic bonds in polysaccharides and contribute to the degradation and remodeling of fungal cell walls. The enzymes belong to the family of auxiliary activities, which catalyze the oxidative cleavage of glycosidic bonds, thereby facilitating the breakdown of complex carbohydrates into simple sugars that fungi can use [88,89]. The presence of these enzymes in various fungal species suggests a robust mechanism for cell wall turnover, similar to the function of LTGs in bacteria. No similar enzyme was identified as a target for RNA editing during sexual reproduction in *N. crassa*. Environmental cues typically influence the production of these lytic enzymes, causing fungi to alter the composition of their cell walls and their processes of degradation. This enables the fungus to enter the plant, receiving nutrients to sustain fungal growth, e.g., within the flower [54,55,90].

As noted above for ascomycete fungi, such as *N. crassa*, during infection by MVLG, in the MILate stage, A-to-I RNA editing takes place in a gene predicted to encode methylenomycin, which, similarly, is an H^+^ antiporter. This gene is involved in maintaining cellular ion homeostasis and moves Na^+^ ions out of the cytosol into the vacuole [56,57,58]. Furthermore, for pathogenic fungi, this methylenomycin: H^+^ antiporter is required for the infection process [91]. Also, this antiporter plays an important role in regulating vacuolar pH, which further indicates its importance in the infection and colonization process of plants by fungi [92]. In different organisms, including plants, it is observed that this type of antiporter can catalyze the exchange of ions across the membrane [93]. Thus, RNA editing of such genes during the MILate stage suggests a significant role in the infection process of fungi and their impact on plant health and growth. Of interest, the MVLG_01566 gene, encoding a putative ADAR, is upregulated in MILate compared with during mating (Supplementary Table S1).

During the FILate stage of MVLG, A-to-I RNA editing occurred in a gene that codes for peptidyl-prolyl cis-trans isomerase (PPIase)-active proteins. These types of proteins are also known as cyclophilins, proteins important for protein folding, assembly, and transport. By facilitating the cis-trans isomerization of Pro residues, these proteins function as molecular chaperones and aid in the proper folding of proteins [94,95,96,97,98]. Within the framework of fungal-plant host–pathogen interactions, cyclophilins have been linked to the correct folding of effector proteins during infection. Furthermore, by stabilizing proteins and membranes under stressful conditions, cyclophilins have been demonstrated to control plant responses to stress, including salt stress [97,99].

In MVLG, one of the genes that was edited in all stages, encoding the Ulp1 protease, showed more editing sites in the mated stage than in the other stages examined. In fungi, Ulp1 is essential for processing the C-terminus of the Smt3 precursor into its mature form by removing its three terminal amino acids [100].

A-to-I editing also occurred in an ABC transporter gene in the case of *N. crassa* during the sexual developmental stage. Additionally, these transporters have contributed to resistance to fungicides and mycotoxins in various fungi [101,102]. The corresponding *Microbotryum* gene was also edited in all stages in both MVLG and MvSup species. That means this ABC-type transporter may be required to adapt in each stage of both fungi for resistance.

Although some similarities were found among preferred target proteins where RNA editing was observed in both the *Microbotryum* species and in ascomycetes (*N. crassa* and *F. graminearum*), some critical differences were seen. In order to explore the sequence context around RNA editing sites in *Microbotryum* during the sexual developmental stage, we found that the distribution of nucleotides within a ±5 nucleotide range centered on each editing site. This method is similar to the one applied in *F. graminearum*, where specific positions like −1 and +1 were found to significantly influence editing efficiency based on the presence of certain preferred nucleotides. While our study did not establish a direct link between nucleotide presence and editing efficiency, our motif analysis consistently showed a probability of cytosine at a preferred position. The preference for cytosine (C) at the −1 position in *Microbotryum* during mating was different from that for uracil (U) at the same position in ascomycetes [10,67]. This suggests that these two groups of fungi exhibit different RNA editing processes. The difference in nucleotide choice suggests that the RNA editing machinery has evolved differently in basidiomycetes (for at least the one example presented here, *Microbotryum*) and ascomycetes; however, additional species need to be examined before broader evolutionary conclusions can be drawn. In fact, we did not discover any orthologs corresponding to the putative adenosine deaminase genes of *Microbotryum* when interrogating the predicted proteomes of *F. graminearum* or *N. crassa* using BLASTp.

A limitation of this study is the use of three biological replicates per condition, which may provide limited statistical power to detect rare, low-level, or stage-specific RNA editing events. Consequently, the editing events identified here are likely to represent the more commonly detected events, whereas studies with larger sample sizes may identify additional rare editing events.

Despite this limitation, in this study, we found that in three distinct *Microbotryum* species, nonsynonymous codon changes occurred more frequently than synonymous changes in all haploid types except one. The substitutions could be related to natural evolutionary processes such as environmental adaptations or specific biological necessities, although their functional and evolutionary significance remain to be determined. Overall, the study of RNA editing in Basidiomycota fungi is a relatively new and evolving field in genetics. Furthermore, the characteristics and mechanisms of RNA editing in fungi are different from those in metazoans. More research is required to fully understand the functional significance and evolutionary implications of RNA editing in fungal biology. The world of *Microbotryum* fungi, with its vast genetic and molecular landscapes, now appears to be a rich field for such scientific discovery.

## Figures and Tables

**Table 1:** Genes uniquely edited in MVLG at the mated stage. log2FC and FDR reflect the pairwise comparison (indicated infection substage vs. mated stage) with the strongest statistical support for each gene (edgeR, FDR < 0.05). Positive values indicate higher expression during infection (lower during mating); negative values indicate the reverse.

Gene ID	Functions	Codon Changes	log2FC (Infection Substage vs. Mated)	FDR	Compared to
MVLG_02763	Subtilase family	Thr681Ala	6.95	2.80 × 10^−88^	MI_8
MVLG_05629	Predicted transporter (major facilitator superfamily)	Tyr12Tyr	−3.87	2.60 × 10^−31^	MILate
MVLG_05664	aspartic-type endopeptidase activity	Ile221Val	7.54	7.20 × 10^−41^	MI_8
MVLG_03646	UNKNOWN	Ser484Pro	−2.5	7.20 × 10^−18^	MI_8
MVLG_05707	oxidoreductase activity	Ala508Ala	−1.47	6.90 × 10^−6^	MILate
MVLG_03240	Tubulin/FtsZ family, GTPase domain	Ser31Ser	8.69	1.30 × 10^−118^	MI_8
MVLG_02490	The nuclear mRNA Export Complex	Gln6Gln	1.52	1.10 × 10^−7^	MI_9
MVLG_06505	F0F1-type ATP synthase, alpha subunit	Gln74Gln	1.52	1.90 × 10^−7^	MI_10
MVLG_01859	Predicted transporter (major facilitator superfamily)	Ile186Thr	6.67	5.40 × 10^−69^	MI_10
MVLG_06406	2OG-Fe(II) oxygenase superfamily	Val256Ala	1.57	3.50 × 10^−6^	MI_9
MVLG_04367	Serine carboxypeptidase S28	Asn144Asp	−2.2	4.50 × 10^−8^	MILate
MVLG_04451	UNKNOWN	Ile35Thr	2.48	6.10 × 10^−9^	MI_10
MVLG_05343	Predicted transporter ADD1 (major facilitator superfamily)	Ser26Pro	1.53	1.70 × 10^−6^	MI_10
MVLG_03076	Helicase of the DEAD superfamily	Val227Ala	5.08	1.30 × 10^−48^	MI_8
MVLG_05391	Magnesium transporter NIPA	Ser1194Pro	−1.69	7.00 × 10^−6^	MI_10
MVLG_05353	UNKNOWN	Asp18Asp	14.93	2.60 × 10^−168^	MI_8
MVLG_07033	UNKNOWN	Leu140Pro	−2.86	2.40 × 10^−12^	MI_8
MVLG_07149	DNA binding	Ser334Pro	−1.8	1.70 × 10^−8^	MILate
MVLG_02891	Putative translation initiation inhibitor UK114/IBM1	Lys91Arg	1.53	7.70 × 10^−9^	MI_8
MVLG_04688	UNKNOWN	Trp87Arg	4.84	3.10 × 10^−46^	MI_10
MVLG_00763	Major Facilitator Superfamily	Leu288Pro	5.18	1.70 × 10^−23^	MI_10
MVLG_07198	DNA replication initiation	Ser579Pro	2.86	3.50 × 10^−15^	MILate
MVLG_06531	UNKNOWN	Ile349Ile	3	9.40 × 10^−8^	MI_FS
MVLG_00922	Predicted transporter (major facilitator superfamily)	Gly206Gly	2.07	1.30 × 10^−8^	MILate
MVLG_01981	UNKNOWN	Ser324Pro	−10.17	2.00 × 10^−11^	MI_10
MVLG_02652	WASP-binding domain of Sorting nexin protein	Gln649Gln	3.42	3.70 × 10^−19^	MILate
MVLG_02745	UNKNOWN	Ser118Pro	−1.77	6.80 × 10^−4^	MI_10
MVLG_03470	UNKNOWN	Trp125Arg	−2.08	1.40 × 10^−7^	MILate
MVLG_06263	DNA binding	Leu100Ser	−4.75	2.10 × 10^−11^	MILate
MVLG_06542	mannan endo-1,4-beta-mannosidase	Trp136Arg	−3.9	1.10 × 10^−8^	MILate
MVLG_06801	UNKNOWN	Ala190Ala	2.14	7.20 × 10^−8^	MILate

**Table 2:** GO Annotation of the edited genes unique in haploids and mating stage of MI.

GO Annotation (M1A1)	GO Annotation (M1A2)	GO Annotation (Mating Stage)
GO:0006508 Proteolysis		GO:0006508 Proteolysis
GO:0006457 Protein folding		GO:0006457 Protein folding
GO:0016491 Oxidoreductase activity	GO:0016491 Oxidoreductase activity	GO:0016491 Oxidoreductase activity
GO:0008270 Zinc ion binding	GO:0008270 Zinc ion binding	GO:0008270 Zinc ion binding
GO:0015074 DNA integration		GO:0015074 DNA integration
GO:0008234 Cysteine-type peptidase activity		GO:0004190 Aspartic-type endopeptidase activity
GO:0003676 Nucleic acid binding	GO:0003676 Nucleic acid binding	GO:0003676 Nucleic acid binding
GO:0008152 Metabolic process	GO:0008152 Metabolic process	GO:0008152 Metabolic process
		GO:0016192 Vesicle-mediated transport
GO:0016272 Prefoldin complex		
GO:0006633 Fatty acid biosynthetic process		
	GO:0035023 Regulation of Rho protein signal transduction	
	GO:0005085 Guanyl-nucleotide exchange factor activity	
	GO:0005515 Protein binding	GO:0005515 Protein binding
		GO:0003968 RNA-dependent RNA polymerase activity
		GO:0051082 Unfolded protein binding
		GO:0055085 Transmembrane transport
	GO:0005509 Calcium ion binding	
	GO:0035556 Intracellular signal transduction	
	GO:0045454 Cell redox homeostasis	
GO:0003824 Catalytic activity	GO:0003824 Catalytic activity	
GO:0005506 Iron ion binding		
	GO:0003779 Actin binding	
		GO:0005524 ATP binding

## Data Availability

Whole-genome shotgun sequencing (WGS), RNAseq data, and genome assemblies generated for this work are available at GenBank BioProject PRJNA1169769. Accession numbers are *M. superbum* (WGS), PRJNA1201796; *M. lychnidis-dioicae* (WGS), PRJNA1202200; *M. intermedium* (WGS), PRJNA1202575. RNASeq data for all experiments listed in this section are available in GEO at NCBI: BioProject PRJNA1169769, Accession Numbers/Record GSE233508 for the *M. superbum* data [24] (Supplementary Table S2) Accession numbers SRX11486121-Accession SRX11486126 for *M. intermedium* data; and BioProject PRJNA246470 for *M. lychnidis-dioicae* data [19,22,24]. The three re-sequenced genomes for each mating type of all three species, done in duplicate, were submitted to NCBI and are available with the following Accession Numbers: *M. superbum* (WGS), PRJNA1201796; *M. lychnidis-dioicae* (WGS), PRJNA1202200; *M. intermedium* (WGS), PRJNA1202575.

## Supplementary Materials

The following supporting information can be downloaded at: https://www.mdpi.com/article/10.3390/jof12090641/s1, Supplementary Table S1. Comparison of Differential expression of Adenosine deaminase Genes between mated stage vs. infection stage (XLSX). Supplementary Table S2. A table with accession numbers and descriptions of strains/growth conditions for all RNA-seq datasets generated or used in this study (XLSX). Supplementary Table S3. Comparison of proportion of A-to-I RNA edits within each species (DOCX). Supplementary Table S4. Genes identified by specific positions, uniquely edited during the mating stage compared to other stages in MvSup. (XLSX). Supplementary Table S5. Functions for unique genes of MILate compared to FILate of MVLG and vice versa. (XLSX). Supplementary Table S6. Genes upregulated in the haploid stage vs. MILate of MVLG. (XLSX). Supplementary Table S7. A table shows the percentage of editing for three examples of genes upregulated in haploids relative to the infection stage (MILate) for MVLG. Here, “1” in the Editing Level column represents 100% editing for the corresponding mRNAs. (XLSX). Supplementary Figure S1. Validation of selected RNA editing sites by cDNA/gDNA PCR and Oxford Nanopore sequencing (PDF). Supplementary Figure S2. Mitogen-activated protein Kinase according to NCBICDS domain identifier (PDF). Supplementary Figure S3. Differential expression level of the genes edited during the MILate stage vs. the haploid stage in different conditions in MVLG (PNG).
