# Supplementary material for: Comparative Analysis of Lifecycle-Dependent A to I RNA Editing in Three Members of the Microbotryum violaceum Fungal Complex
Source: J Fungi (Basel). 2026 Aug 28;12(9):641. doi: 10.3390/jof12090641 (PMC13608869; doi:10.3390/jof12090641)
Supplement: Supplementary file 1 [file jof-12-00641-s001.zip › Supplementary Figure S1.pdf]

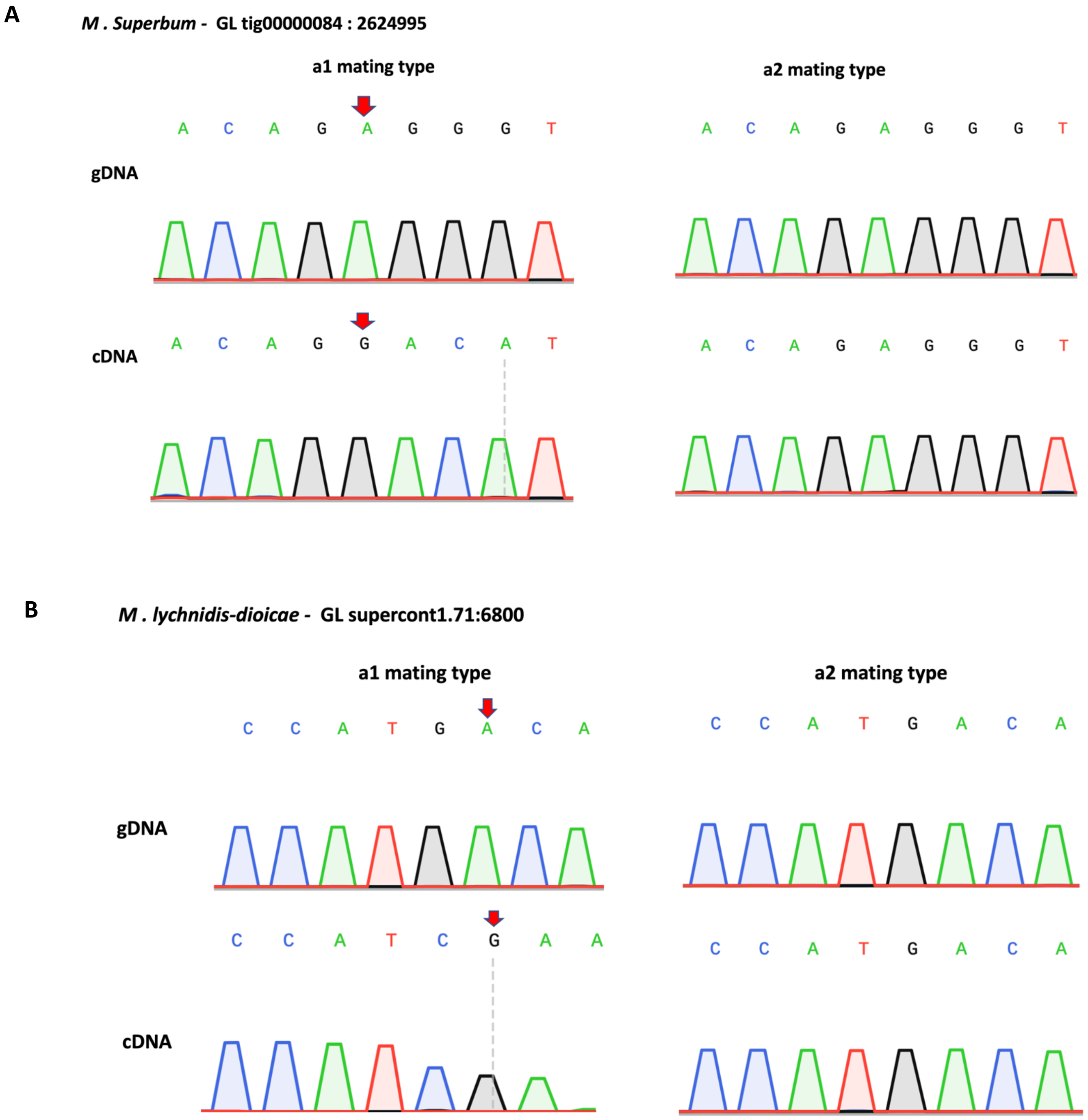
A
M . Superbum - GL tig00000084 : 2624995
a1 mating type
a2 mating type
gDNA
A C A G A G G G T
A C A G A G G G T
cDNA
A C A G G A C A T
A C A G A G G G T
B
M . lychnidis-dioicae - GL supercont1.71:6800
a1 mating type
a2 mating type
gDNA
C C A T G A C A
C C A T G A C A
cDNA
C C A T C G A A
C C A T G A C A

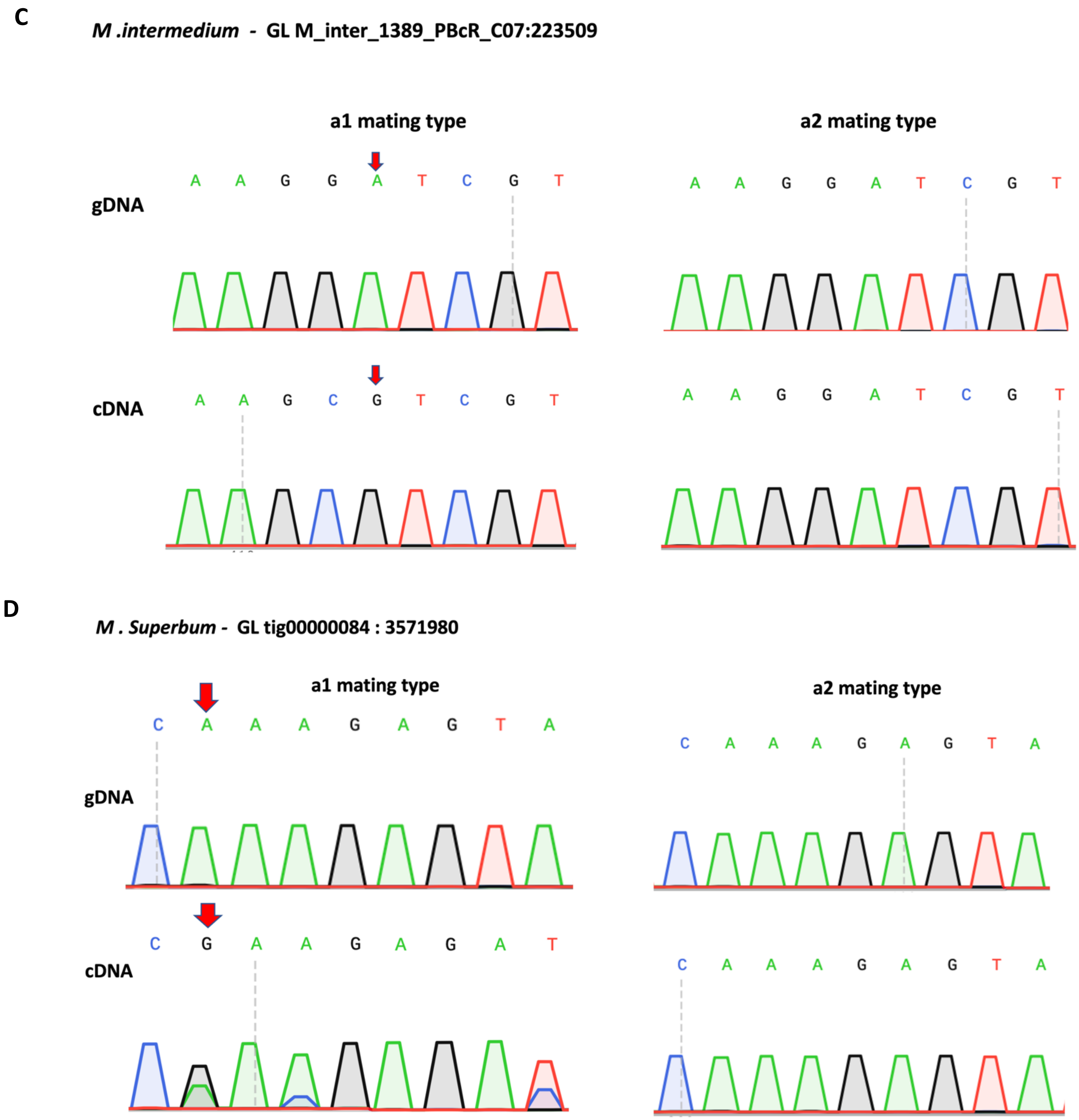

Figure S1. Validation of putative RNA editing sites by PCR amplification and Oxford Nanopore sequencing. Putative editing sites identified from the RNA editing analysis were PCR-amplified and subjected to Oxford Nanopore sequencing for validation. (A) *Microbotryum superbum*, GL tig00000084:2624995, showing 100% editing level. (B) *M. lychnidis-dioicae*, GL supercont1.71:6800, showing 100% editing level. (C) *M. intermedium*, GL M_inter_1389_PBcR_C07:223509, showing 100% editing level. (D) *M. superbum*, GL tig00000084:3571980, showing approximately 70% editing level.
