# Supplementary material for: Comparative Analysis of Lifecycle-Dependent A to I RNA Editing in Three Members of the Microbotryum violaceum Fungal Complex
Source: J Fungi (Basel). 2026 Aug 28;12(9):641. doi: 10.3390/jof12090641 (PMC13608869; doi:10.3390/jof12090641)
Supplement: Supplementary file 1 [file jof-12-00641-s001.zip › Supplementary Table S3.pdf]

**Mating-type comparison**	**Mean proportion**	**95% confidence interval**
6P vs. 6D	0.4915	0.456–0.527
p1A1 vs. p1A2	0.2147	0.192–0.238
MIA1 vs. MIA2	0.8214	0.762–0.880
**Species comparison**	**z-ratio**	**Tukey-adjusted p-value**
MvSup vs. MI	−8.03	<0.0001
MvSup vs. MVLG	12.69	<0.0001
MI vs. MVLG	17.14	<0.0001
